# *BdCESA7*, *BdCESA8*, and *BdPMT* Utility Promoter Constructs for Targeted Expression to Secondary Cell-Wall-Forming Cells of Grasses

**DOI:** 10.3389/fpls.2016.00055

**Published:** 2016-02-04

**Authors:** Deborah L. Petrik, Cynthia L. Cass, Dharshana Padmakshan, Cliff E. Foster, John P. Vogel, Steven D. Karlen, John Ralph, John C. Sedbrook

**Affiliations:** ^1^School of Biological Sciences, Illinois State University, NormalIL, USA; ^2^U.S. Department of Energy Great Lakes Bioenergy Research Center, University of Wisconsin–Madison, MadisonWI, USA; ^3^U.S. Department of Energy Great Lakes Bioenergy Research Center, Michigan State University, East LansingMI, USA; ^4^U.S. Department of Energy Joint Genome Institute, Walnut CreekCA, USA; ^5^Department of Biochemistry, Wisconsin Energy Institute, University of Wisconsin–Madison, MadisonWI, USA

**Keywords:** binary vectors, *Brachypodium*, cellulose, lignin, monocot, *p*-coumarate, tissue-specific expression

## Abstract

Utility vectors with promoters that confer desired spatial and temporal expression patterns are useful tools for studying gene and cellular function and for industrial applications. To target the expression of DNA sequences of interest to cells forming plant secondary cell walls, which generate most of the vegetative biomass, upstream regulatory sequences of the *Brachypodium distachyon* lignin biosynthetic gene *BdPMT* and the cellulose synthase genes *BdCESA7* and *BdCESA8* were isolated and cloned into binary vectors designed for *Agrobacterium*-mediated transformation of monocots. Expression patterns were assessed using the β-glucuronidase gene *GUSPlus* and X-glucuronide staining. All three promoters showed strong expression levels in stem tissue at the base of internodes where cell wall deposition is most active, in both vascular bundle xylem vessels and tracheids, and in interfascicular tissues, with expression less pronounced in developmentally older tissues. In leaves, *BdCESA7* and *BdCESA8* promoter-driven expression was strongest in leaf veins, leaf margins, and trichomes; relatively weaker and patchy expression was observed in the epidermis. *BdPMT* promoter-driven expression was similar to the *BdCESA* promoters expression patterns, including strong expression in trichomes. The intensity and extent of GUS staining varied considerably between transgenic lines, suggesting that positional effects influenced promoter activity. Introducing the *BdPMT* and *BdCESA8* Open Reading Frames into *BdPMT* and *BdCESA8* utility promoter binary vectors, respectively, and transforming those constructs into *Brachypodium pmt* and *cesa8* loss-of-function mutants resulted in rescue of the corresponding mutant phenotypes. This work therefore validates the functionality of these utility promoter binary vectors for use in *Brachypodium* and likely other grass species. The identification, in *Bdcesa8-1* T-DNA mutant stems, of an 80% reduction in crystalline cellulose levels confirms that the *BdCESA8* gene is a secondary-cell-wall-forming cellulose synthase.

## Introduction

Efforts are underway to engineer plant vegetative biomass, such as stems and leaves of grasses, to be more easily deconstructed and converted to liquid biofuels such as ethanol (Carroll and Somerville, 2009). Since secondary cell walls make up most of the vegetative biomass in mature plants, they are the major target for efforts to improve the efficiency of biofuel production. For acidic and alkaline pretreatment-based conversion processes, the highly recalcitrant phenolic polymer lignin poses challenges in that it encapsulates cellulose and perhaps crosslinks to hemicelluloses thereby interfering with their chemical and enzymatic hydrolysis (Chapple et al., 2007; Chen and Dixon, 2007). One approach to improving biomass deconstructability is to engineer genetic changes that alter flux through the lignin biosynthetic pathway, by knocking down or overexpressing key monolignol biosynthetic pathway genes resulting in lower lignin quantity, altered lignin composition, and/or altered cell wall polymer crosslinking (Vanholme et al., 2012; Eudes et al., 2014; Wilkerson et al., 2014).

Another approach to improve plant biomass yields and deconstruction properties is to introduce genetic changes that either increase deposition of secondary cell wall polysaccharides or result in the deposition of less tightly packed (amorphous) polysaccharides. However, when transgenes that alter these traits are expressed in a non-targeted fashion, they can have detrimental effects on plant health. Examples include collapsed xylem elements or stem lodging, developmental abnormalities, or toxicity that negatively affects plant growth and fitness (Yang et al., 2013). The creation of a toolbox of directed gene promoters that drive expression of transgenes in cells involved in secondary cell wall formation would be of benefit to researchers aiming to improve plant biomass properties for biofuels generation while minimizing plant fitness costs.

Bioinformatic resources are now available for *Brachypodium distachyon* (Brachypodium) that allow its transcriptome to be mined for genes having various expression patterns^1^ (e.g. Mutwil et al., 2011). Coussens et al. (2012) searched the Brachypodium Expressed Sequence Tag (EST) database^2^ for genes that were either constitutively expressed across tissues and development, or uniquely expressed in leaf, stem plus leaf sheaths, roots, flowers, and/or callus. They demonstrated that a combined approach of bioinformatic purveyance of all expressed genes and whittling down of promoter candidates based on qRT-PCR analysis, followed by promoter-GUS fusion analyses *in planta*, was effective in identifying gene promoters with desired expression patterns for a particular transgenic application.

Transcriptome profiling was also employed to determine the tissue expression patterns of the ten-member Brachypodium *CELLULOSE SYNTHASE A* (*CESA*) gene family (Handakumbura et al., 2013). *CESA4* (Bradi3g28350)*, CESA7* (Bradi4g30540), and *CESA8* (Bradi2g49912) were determined to be expressed at high levels in plant organs known to be active in secondary cell wall biosynthesis. The expression patterns of *CESA4* and *CESA7* were determined by *in situ* hybridization to be highest in vascular bundles, surrounding interfascicular fibers, and epidermal cells (Handakumbura et al., 2013). Artificial microRNA knockdown of *CESA4* and *CESA7* in Brachypodium plants resulted in reduced secondary cell wall thickness and crystalline cellulose amounts, thereby demonstrating that those two genes encode cellulose synthase catalytic subunits involved in synthesizing cellulose deposited in secondary cell walls (Handakumbura et al., 2013).

An elegant example of how targeted tissue-specific gene expression can improve plant biomass as a feedstock for biofuels generation can be found in Yang et al. (2013). In that study, *CINNAMATE 4-HYDROXYLASE* (*C4H*) gene activity reintroduced into an *Arabidopsis c4h* mutant background under the control of the xylem vessel-specific *VASCULAR-RELATED NAC-DOMAIN6* (*VND6*) gene promoter directed normal lignin production to xylem vessels. Cell wall strength was maintained and vessel element collapse avoided, while lignin amounts remained reduced in interfascicular fiber cells, thus allowing for easier extraction of polysaccharide sugars. This example provides evidence that promoter substitution can successfully alter cell wall phenotypes including lignin deposition while avoiding negative growth effects.

Recently, the *Populus trichocarpa* (poplar) *PtrCesA8* promoter was used to drive expression of the R2R3 MYB transcription factor *PtrMYB152* in *Arabidopsis thaliana* resulting in the upregulation of secondary cell wall biosynthetic genes and thicker cell walls (Wang et al., 2014). In a study particularly promising to biofuels applications, the *PtrCesA8* promoter was used in poplar to drive expression of the *Angelica sinensis FERULOYL MONOLIGNOL TRANSFERASE* (*AsFMT*) gene (Wilkerson et al., 2014). The AsFMT enzyme functions to acylate sinapyl or coniferyl monolignol alcohols with ferulate, producing ferulate-monolignol ester conjugates that were incorporated by radical oxidative coupling into the backbone of the growing lignin polymer. Due to the facility of breaking this ester bond, lignin modified in this way has been termed “zip-lignin”. This technology could lower the cost of biomass pretreatment due to a lesser requirement for high heat or chemical treatments (Wilkerson et al., 2014).

For the present study, the upstream regulatory sequences of the Brachypodium *p-COUMAROYL-CoA MONOLIGNOL TRANSFERASE* (*BdPMT*), *BdCESA7*, and putative *BdCESA8* cellulose synthase genes were cloned in order to generate utility promoter cassettes functional in grasses for targeting gene-of-interest expression in secondary-cell-wall-forming cells. These promoters were chosen because the corresponding genes have been implicated in lignin biosynthesis (*BdPMT*; Petrik et al., 2014) and secondary-cell-wall cellulose biosynthesis (*BdCESA7* and *BdCESA8*; Handakumbura et al., 2013). *Brachypodium* plants transformed with each of these utility promoter vector constructs, into which the *GUSPlus* reporter gene was introduced, displayed GUS staining patterns that would be expected for promoters involved in secondary cell wall formation. In addition, functionality of the utility promoter cassettes was validated by rescuing the mutant phenotypes of the previously reported *Bdpmt-1* loss-of-function mutant (Petrik et al., 2014) and a newly identified *Bdcesa8-1* T-DNA mutant by transforming into those mutants the *BdPMT* and *BdCESA8* coding sequences inserted into the *BdPMT* and *BdCESA8* promoter constructs, respectively. These utility promoter vectors should have utility not only in Brachypodium but also in other monocots including cereals and dedicated bioenergy crop grasses.

## Materials and Methods

### Vector Construction

The monocot transformation vector pIPKb001 (Himmelbach et al., 2007) was the backbone vector sequence for constructing the utility promoter vectors (maps shown in **Supplementary Figures S1**–**S3**) and subsequent *GUSPlus* (Cambia Labs) expression vectors. Initially, the *Zea mays UBIQUITIN1* intron (*ZmUbi1IN*) was PCR-amplified from the pStarling shuttle vector (Gubler et al., 2008) using ZmUbiIN1AscI_F and ZmUbiIN1HindIII_R primers and ligated into the AscI and HindIII restriction sites of the multiple cloning site (MCS) of pIPKb001. Next, *BdPMT* (Bradi2g36910), *BdCESA7* (Bradi4g30540), or *BdCESA8* (Bradi2g49912) promoter sequences were PCR-amplified from *Brachypodium* inbred line Bd21-3 genomic DNA, using primer pairs BdPMTproSwaI_F and BdPMTproAscI_R, BdCESA7proSwaI_F and BdCESA7proAscI_R, or BdCESA8proStuI_F and BdCESA8proAscI_R, respectively. PCR products were digested with either SwaI and AscI (*BdPMT* and *BdCESA7*) or StuI and AscI (*BdCESA8*), and ligated into the corresponding MCS sites in pIPKb001 upstream of the *ZmUbi1IN*. The 3,000 nt *BdPMT* promoter fragment consisted of sequence from (–) 3044 to (–) 45 relative to the translational start site of BdPMT, Bd2:37,332,132-37,335,133 reverse from the *Brachypodium distachyon* v2.1 genomic sequence database^3^ (Phytozome v10.3). Similarly, the 2,385 nt *BdCESA7* promoter fragment consisted of sequence from (–) 2385 to (–) 1 relative to the translational start site of BdCESA7 (Bd4:36,373,412-36,375,796), and the 1,888 nt *BdCESA8* promoter fragment spanned sequence from (–) 1888 to (–) 1 relative to the translational start site of BdCESA8 (Bd2:49,946,427-49,948,277). Sequence analysis found that the Bd21-3-derived *BdCESA8* promoter sequences had minor sequence differences compared to the Joint Genome Initiative (JGI)-generated Bd21 reference genome sequences.

To generate the GUS reporter gene constructs, *GUSPlus* was PCR-amplified from the pGPro8 vector (Genbank accession JN593327.1) using the primers GUSPLUS_BamHI_F and GUSPLUS_XhoI_R, cloned into the BamHI and XhoI sites of pENTR2B, then inserted into the utility promoter vectors using Gateway-cloning technology.

The *BdPMTprom::BdPMT ORF* phenotypic rescue construct was generated by PCR-amplifying the *BdPMT* (Bradi2g36910) Open Reading Frames (ORF) from *Brachypodium* inbred line Bd21-3 first-strand cDNA using the primer pair BdPMTBamHI_F and BdPMTSpeIXhoI_R, restriction digesting the PCR product with BamHI and XhoI and ligating into pENTR2B, then Gateway-cloning into the *BdPMT* utility promoter vector described above.

The *BdCESA8prom::BdCESA8 ORF* phenotypic rescue construct (map shown in **Supplementary Figure S11**) was generated by PCR-amplifying the *BdCESA8* (Bradi2g49912) ORF from first-strand cDNA using the primer pair BdCESA8SalI_F and BdCESA8XhoI_R, restriction digesting the PCR product with SalI and XhoI and ligating into pENTR2B, then Gateway-cloning into the *BdCESA8* utility promoter vector. The *BdCESA8prom::BdCESA8 ORF* cassette (composed of the *BdCESA8* promoter fragment, *ZmUbi1IN*, Gateway attB1 and attB2 recombination sites flanking the *BdCESA8 ORF*, and the NOS terminator) was then released by SfiI restriction enzyme digestion and ligated into the custom-made B258 p9ioACT vector carrying a paromomycin selection cassette^4^ (DNA Cloning Service).

PCR primers are listed in **Supplementary Table S1**. All inserts were verified by sequencing. Vector sequences have been deposited into Genbank (accession numbers KT948986, KT948987, and KT962835 for the *BdPMT*, *BdCESA7*, and *BdCESA8* utility promoter vectors, respectively).

### *Agrobacterium tumefaciens*-Mediated Transformation

Plants harboring the *BdPMTprom::GUSPlus, BdCESA7prom::GUSPlus, or BdCESA8prom::GUSPlus* constructs were regenerated from Bd21-3 embryonic callus by *Agrobacterium* strain AGL-1 mediated transformation (Vogel and Hill, 2008) using media supplemented with 40 U/mL hygromycin B (Phytotechnology Laboratories). Similarly, plants harboring the *BdPMTprom::BdPMT ORF* rescue construct were regenerated from *Bdpmt-1* (Petrik et al., 2014) embryonic callus on media supplemented with hygromycin as above. Plants harboring the *BdCESA8prom::BdCESA8 ORF* rescue construct were regenerated from callus originating from embryos dissected from heterozygous *BdCESA8 / BdcesA8-1* plants (homozygous *BdcesA8-1* plants could not be used because they were sterile). The *Agrobacterium*-transformed callus was cultured on media supplemented with 120 mg/L paromomycin (PhytoTechnology Laboratories). The *BdcesA8-1* mutant is a T-DNA line, JJ18282, harboring a T-DNA insertion in the 9th exon of Bradi2g49912. JJ18282 was obtained from the WRRC *Brachypodium distachyon* T-DNA collection (Bragg et al., 2012) that is now housed at the DOE Joint Genome Institute^5^ Primers used for genotyping are listed in **Supplementary Table S1** and their relative locations shown in **Figure 7C.**

### Seed Sterilization and Plant Growth

Transgenic seeds were surface-sterilized and plated on selective agar supplemented with either 40 U/mL hygromycin B (Phytotech Labs) or 120 mg/L paromomycin, stratified for 3 days at 4°C in the dark, then moved to a 22°C growth chamber under 16 h light for 5–7 days. Seedlings were transplanted to a 50:50 mix of SunGro Rediearth and MetroMix 510 soil in 10 cm pots, and allowed to grow and senesce in a growth chamber under a 20 h light:4 h dark photoperiod at 22°C and 50% humidity. Control plants were either wild-type inbred line Bd21-3 seedlings plated on non-selective plates or planted directly into soil, or hygromycin-selected plants harboring a *Zea mays UBIQUITIN1* (*ZmUbi1*) promoter with intron (*ZmUbi1IN*) driving *GUSPlus* (Cambia Labs) in pWBVec8 (Wang et al., 1998).

### Tissue Collection and GUS Staining

For leaf expression analyses, 2 cm distal sections were taken from the top two leaves of plants 23 and 37 days after planting (DAP). For stem expression analyses, transverse hand cut sections were taken at the median of every internode, except for the apical internode that was sectioned within the basal 2 cm, at 23 or 37 DAP. Floral structures were obtained just before anthesis when neighboring distal florets were undergoing seed fill. Root tips from seedlings germinated on plates were excised for root expression analyses.

The expression pattern for each promoter in leaves, stems, and flower structures was determined by colorimetric GUS staining by immersion in 0.1 M NaPO_4_ pH 7.0/10 mM EDTA pH 8.0/0.1% (v/v) Triton X-100/2 mM 5-bromo-4-chloro-1*H*-indol-3-yl β-D-glucopyranosiduronic acid (X-Gluc) dissolved in *N,N*-dimethyl formamide (Jefferson, 1987). Roots were stained similarly, with the addition of 10 mM ascorbic acid to prevent browning. Samples were incubated overnight at either 25°C (flowers) or 37°C (leaves, stems, and roots). Additionally, tissues isolated from lines with the highest expression levels were also stained in the presence of potassium 1 mM each ferri- and ferrocyanide. Post-staining, leaves were cleared in 70% ethanol, and all samples were rinsed in water before imaging using brightfield microscopy.

### *p*-Coumaric Acid and Crystalline Cellulose Quantitation

The *p*-coumarate content of ball-milled, extractive-free stem plus leaf sheath cell wall material from senesced plants was determined by alkaline hydrolysis to release *p*-coumaric acid (*p*CA) that was quantitated by Gas Chromotography and Flame Ionization Detection (GC-FID) as described in Ralph et al. (1994).

The crystalline cellulose content of ball-milled, extracted, and destarched stem plus leaf sheath alcohol-insoluble residue (AIR) cell wall material from senesced stems plus leaf sheaths was determined by sulfuric acid hydrolysis as described previously (Selvendran and O’Neill, 1987; Foster et al., 2010). The resulting monosaccharide (glucose) was quantitated using a colorimetric anthrone assay.

## Results

### Generation of the *BdPMT*, *BdCESA7*, and *BdCESA8* Utility Promoter Vectors

Previously we showed that the *Brachypodium BdPMT* gene catalyzes the formation of monolignol *p*-coumarate (ML-*p*CA) ester conjugates that become polymerized into lignin within secondary cell walls (Petrik et al., 2014). Therefore, we reasoned that the promoter and *cis*-acting regulatory sequences of the *BdPMT* gene would be useful for driving the expression of gene knockdown constructs or genes of interest, with spatial and temporal patterns associated with lignin deposition. In addition, utility promoters from *CESA* genes of dicot species have proven useful for targeting expression to cells producing cellulose in their secondary cell walls (Wang et al., 2014; Wilkerson et al., 2014), so we set out to generate equivalent secondary-cell-wall-associated *CESA* utility promoters that would be fully functional in grasses.

In order to clone *BdPMT*, *BdCESA7*, and *BdCESA8* DNA fragments containing each gene’s 5′ untranslated region (UTR), promoter, and upstream regulatory sequences (hereafter referred to as promoter fragments), *Brachypodium* inbred line Bd21-3 genomic DNA was used as a template along with sequence-specific primers (**Supplementary Table S1**) to PCR-amplify a 3,000 bp fragment located upstream of the *BdPMT* (Bradi2g36910) gene translational start site as well as 2,385 and 1,888 bp fragments located immediately upstream of the *BdCESA7* (Bradi4g30540) and *BdCESA8* (Bradi2g49912) genes’ translational start sites, respectively. The promoter fragments were then cloned, by restriction digestion and ligation, into the pIPKb001 monocot binary vector (Himmelbach et al., 2007) immediately upstream of a maize *UBIQUITIN1* intron (*ZmUbi1IN*) that was inserted into unique AscI/HindIII restriction sites within the vector’s multiple cloning site (**Supplementary Figures S1**–**S3**). The *ZmUbi1IN* was introduced because it has been reported that an intron fused to the 5′ end of gene-of-interest coding sequences improved exogenous gene expression in monocot species (Mascarenhas et al., 1990; Plesse et al., 2001; Sivamani and Qu, 2006; Mann et al., 2011).

These assembled constructs are the so-called utility promoter vectors, the maps of which are shown in **Supplementary Figures S1**–**S3**. The corresponding DNA sequences can be found in Genbank (Accession numbers KT948986, KT948987, and KT962835). Each of the vectors contains unique SfiI restriction sites located immediately upstream of each promoter and immediately downstream of the *NOPALINE SYNTHASE* (*NOS*) translational terminator sequences (*NOS* terminator). As such, each so-called promoter cassette, which is composed of the promoter fragment, *ZmUbi1IN*, Gateway attR1 and attR2 recombination sites flanking the chloramphenicol resistance (*CmR*) and *ccdB* genes, and the *NOS* terminator, can be released by Sfi1 restriction enzyme digestion and moved to, e.g., another binary vector harboring a plant selectable marker other than the *HYGROMYCIN PHOSPHOTRANSFERASE* (*HPT*) gene present in these utility promoter vectors. This, in fact, was done as part of our effort to rescue the *Bdcesa8-1* T-DNA insertional mutant phenotypes (described below); the *BdCESA8* promoter cassette, composed of the *BdCESA8* promoter fragment, *ZmUbi1IN*, Gateway attB1 and attB2 recombination sites flanking the *BdCESA8 ORF*, and the *NOS* terminator, was moved into the B258 p9ioACT vector containing the *NEOMYCIN PHOSPHOTRANSFERASE II* (*NPTII*) gene, given that the *HPT* gene was already present in *Bdcesa8-1* plants within the T-DNA insertion disrupting the *BdCESA8* gene.

In order to assess the gene expression patterns that these promoter cassettes could drive *in planta*, the *GUSPlus* reporter gene was Gateway-cloned into the attR1 and attR2 recombination sites of the three utility promoter vectors, thereby replacing the *CmR* and *ccdB* genes. Each construct was then transformed into *Brachypodium* embryo-derived callus using *Agrobacterium tumefaciens* followed by regeneration of T_0_-generation transgenic plants on hygromycin selection media. Four independent T_0_-generation *BdPMTprom::GUSPlus* plants were generated along with 20 and 33 independent T_0_-generation *BdCESA7* and *BdCESA8prom::GUSPlus* plants, respectively.

### The *BdPMTprom::GUSPlus* Expression Pattern was Comparable to the Phloroglucinol Staining Pattern of Lignin

**FIGURE 1:**
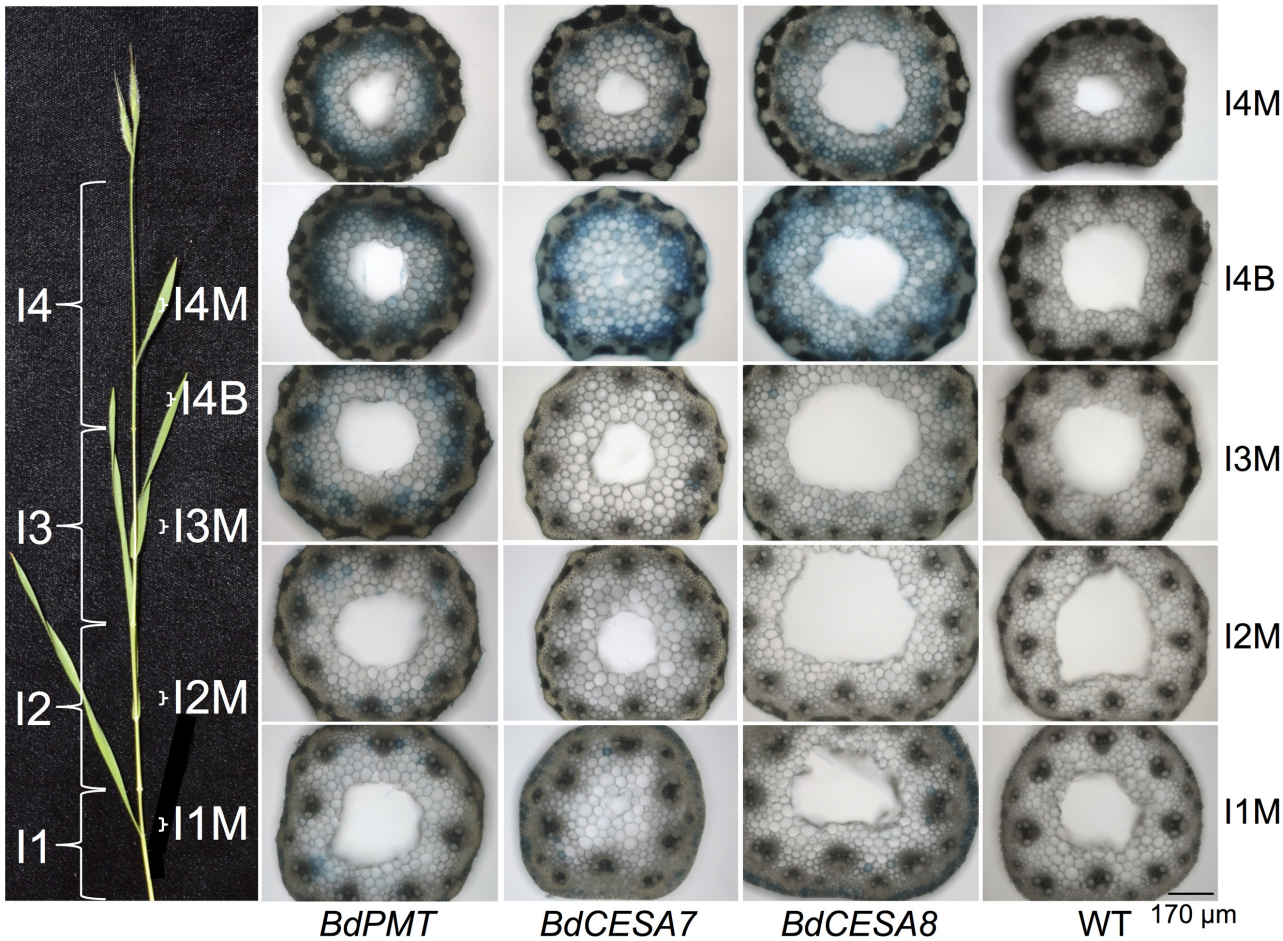
**GUS-stained *Brachypodium distachyon* stem cross sections from 37-day-old plants transformed with the *BdPMT* (first column), *BdCESA7* (second column), and *BdCESA8prom::GUSPlus* (third column) constructs.** Wild type (WT) control sections are shown in the fourth column. Sections for a given line were from the same culm. All sections were stained the same duration so as to show relative expression levels. Pictured on the left is a culm with its internodes (I) labeled along with the locations from which each section was taken. B = base and M = middle of internode. Scale bar for sections = 170 μm.

**FIGURE 2:**
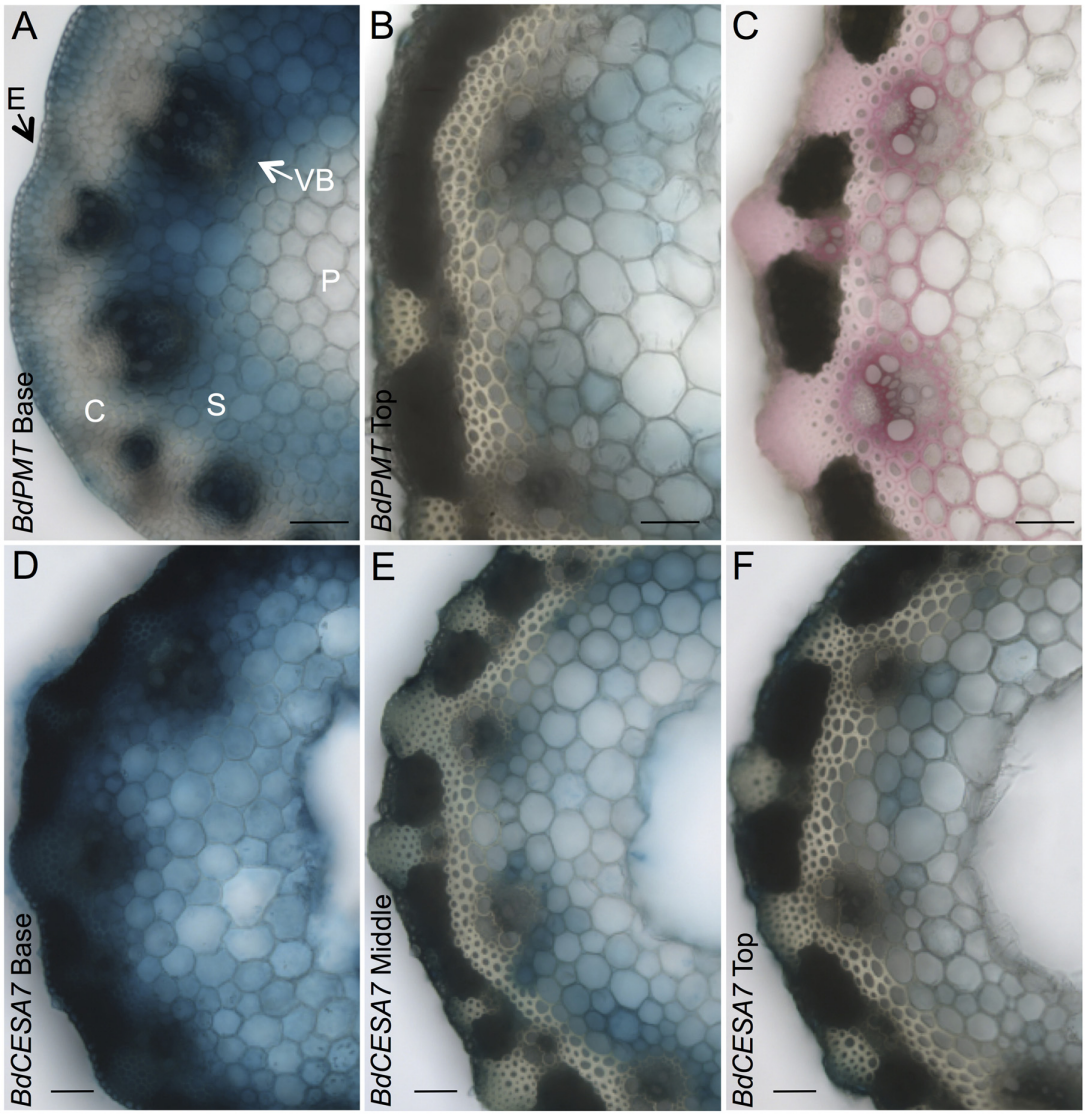
**GUS- and phloroglucinol-stained stem cross sections from 37-day-old plants.** **(A,B)** *BdPMTprom::GUSPlus* sections from the base **(A)** and top **(B)** of the apical internode. **(C)** Stem section from the top of an apical internode stained with phloroglucinol to visualize lignin (red coloration). **(D–F)** *BdCESA7prom::GUSPlus* sections from the base **(D)**, middle **(E)**, and top **(F)** of the apical internode. Sections for a given line were from the same internode. All sections were GUS-stained the same duration so as to show relative expression levels. Note that some of blue color in the pith may be from diffusion of the colorimetric product. E = Epidermis; C = Cortex; VB = Vascular Bundle; S = Sclerenchyma; P = Parenchyma. Scale bar = 50 μm.

**FIGURE 3:**
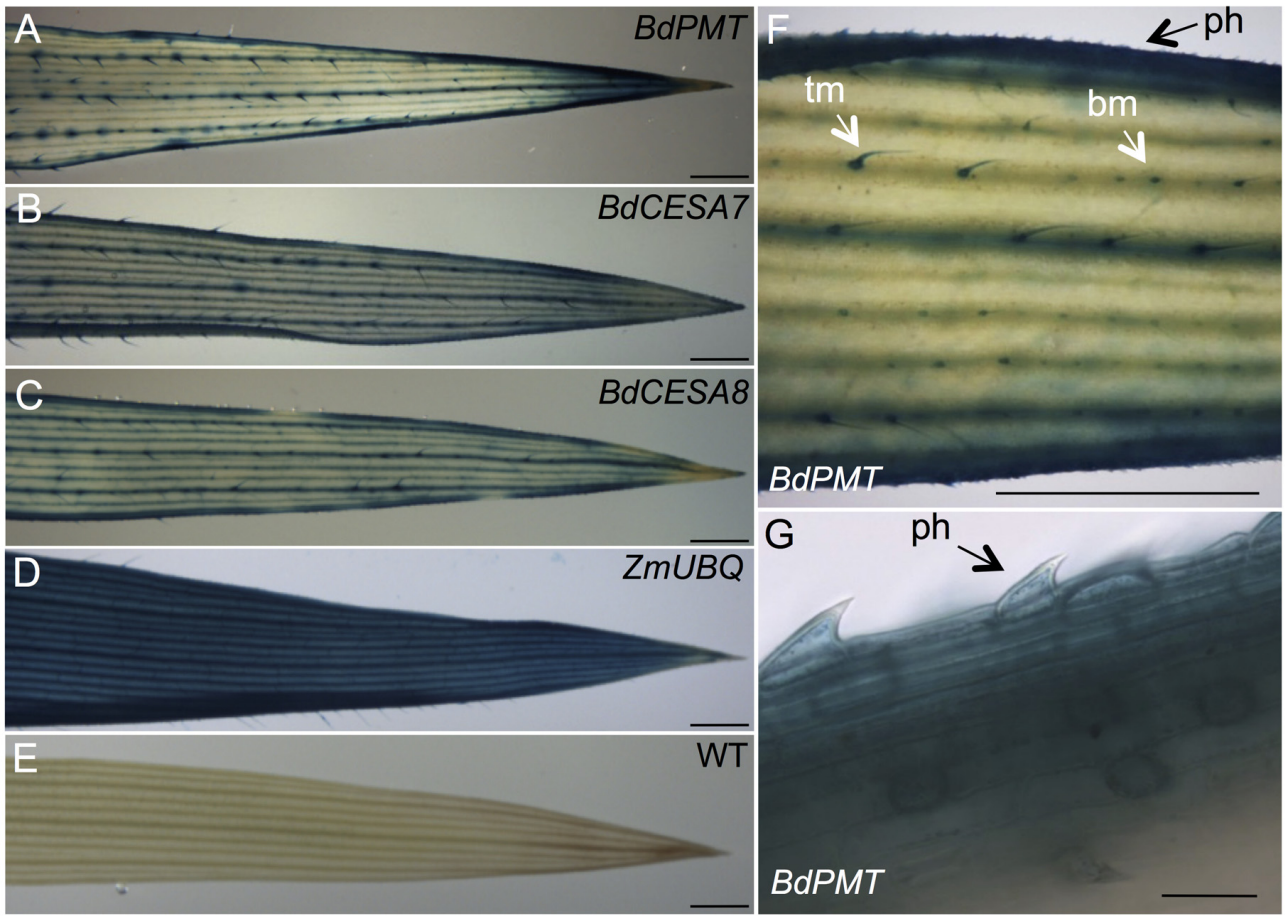
**GUS-stained leaf snippets from 37-day-old plants.** **(A)** *BdPMTprom::GUSPlus.* **(B)** *BdCESA7prom::GUSPlus*. **(C)** *BdCESA8prom::GUSPlus*. **(D)** *ZmUBQprom::GUSPlus*. **(E)** Wild type. **(F,G)** Close-up views of GUS-stained *BdPMTprom::GUSPlus* leaves. ph = prickle hair. Tm = trichome macrohair; bm = bicellular microhair. Scale bars in **A–F** = 1 mm. Scale bar in **G** = 30 μm.

**FIGURE 4:**
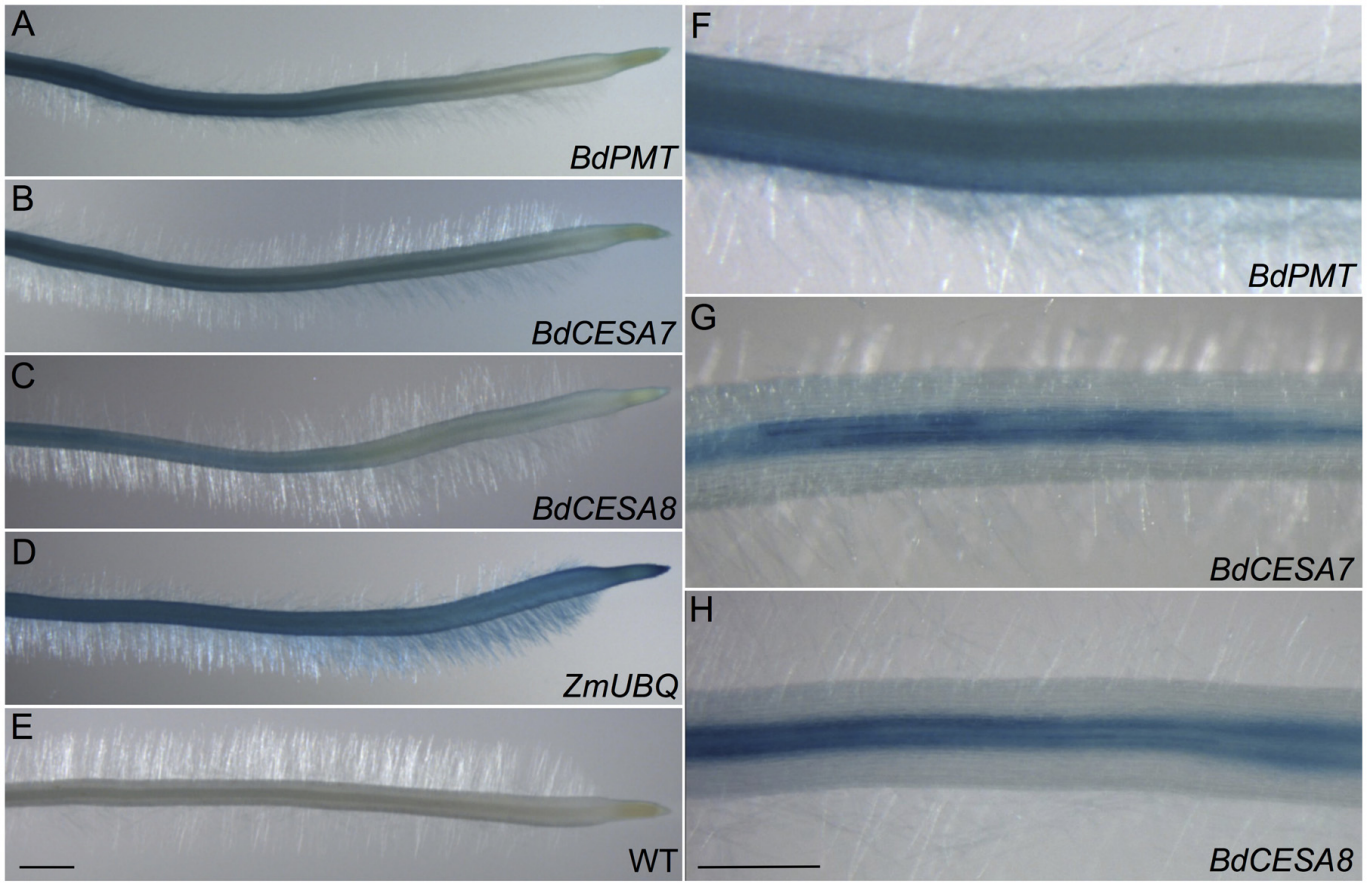
**GUS-stained roots from 3-day-old seedlings.** **(A,F)** *BdPMTprom::GUSPlus.* **(B,G)** *BdCESA7prom::GUSPlus*. **(C,H)** *BdCESA8prom::GUSPlus*. **(D)** *ZmUBQprom::GUSPlus*. **(E)** Wild type. Scale bar = 1 mm for **A–E**, 0.5 mm for **F–H**.

**FIGURE 5:**
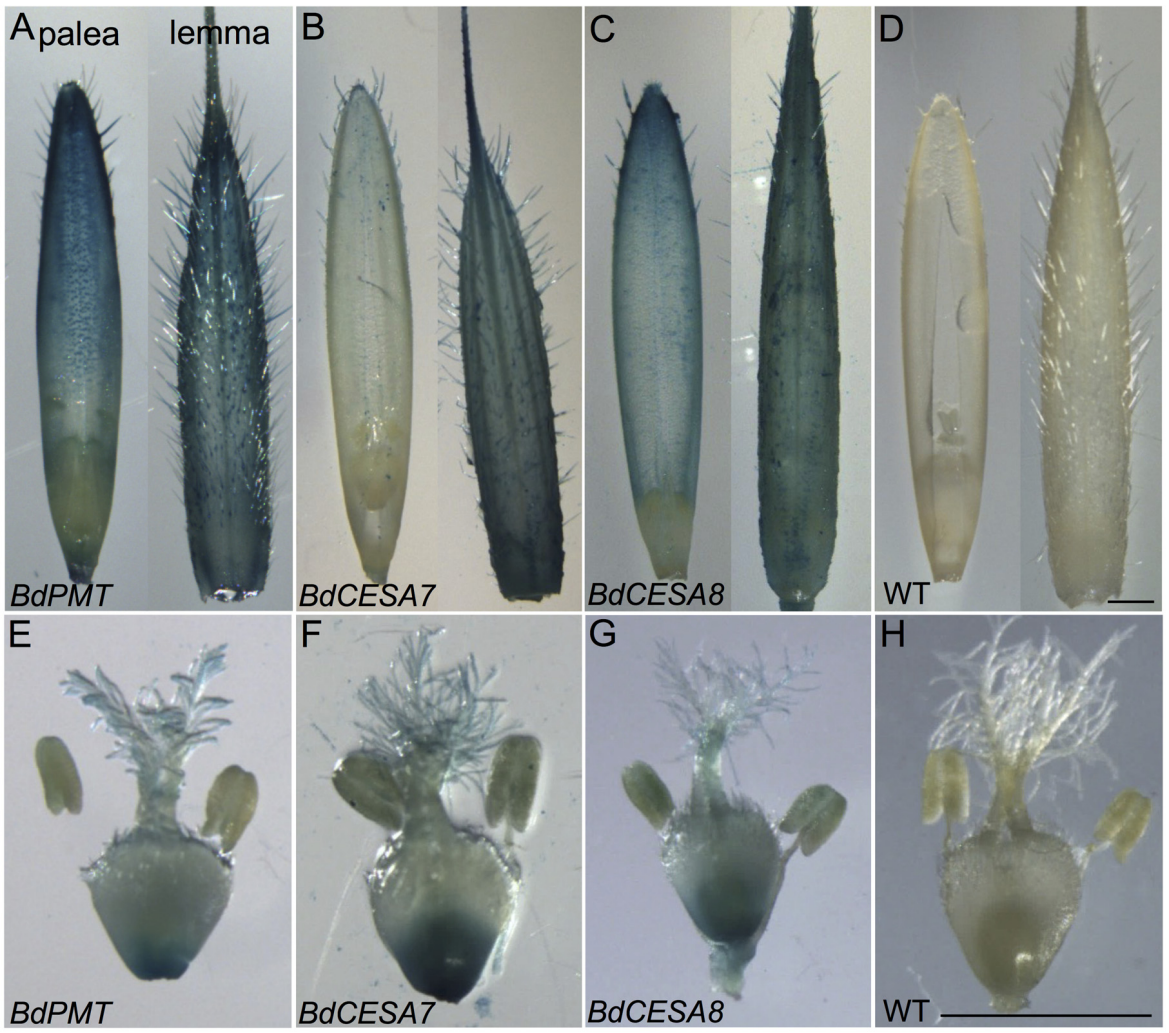
**GUS-stained florets and internal floral organs prior to seed fill.** **(A,E)** *BdPMTprom::GUSPlus.* **(B,F)** *BdCESA7prom::GUSPlus*. **(C,G)** *BdCESA8prom::GUSPlus*. **(D,H)** Wild type. Shown in **(A–D)** are the palea (left) and lemma (right). Shown in **(E–H)** are the pistil and anthers. Scale bars = 1 mm.

T_1_- and T_2_-generation plants from four independent transgenic lines harboring the *BdPMTprom::GUSPlus* construct were analyzed for X-Glucuronide (GUS) staining in seedlings and plants at different developmental stages (**Figures 1–5**; **Supplementary Figures S4**–**S6**, **S9**, **S10**). In 37-day-old adult plant stems, GUS staining was observed in sections taken from all internodes, with staining being strongest in the developmentally younger internodes at the top of the culm (**Figure 1**). Within an internode, staining was strongest at the base where tissues are developmentally the youngest and are actively forming secondary cell walls (**Figure 2A**). In sections from the internode base, GUS staining was strongest in vascular bundles, particularly in the xylem vessels and tracheids, as well as in interfascicular tissues, which contain sclerenchyma cells. Strong GUS staining was also visible in epidermal cells including in microhair trichomes, with indigo product extending into the chlorenchyma and sclerenchyma cells comprising the cortex (**Figure 2A**). The reader is referred to Matos et al. (2013) for illustrations showing the locations of different cell and tissue types in *Brachypodium* stem sections.

In stem sections taken from the middle and top of internodes, *BdPMTprom::GUSPlus*-derived GUS staining was most noticeable in interfascicular tissue cells, being much weaker compared to that observed in sections taken from the base (**Figures 2A,B**). These differences in relative intensities of expression were expected, given that the youngest and developmentally most active tissue is found at the base of internodes near the intercalary meristems (Esau, 1965). In strongly expressing lines, short incubation times or addition of ferri- and ferrocyanide resulted in patterns similar to those of weakly expressing lines. Potassium ferri- and ferrocyanide reduces colorimetric product diffusion (Vitha et al., 1995).

Stem-section GUS staining patterns correlated with where lignin was detected using phloroglucinol staining (**Figure 2C**). It is worth noting that phloroglucinol staining depicts how much lignin has accumulated over time whereas GUS staining depicts gene expression at that particular moment of stem development.

The tissues of 23-day-old plants were also analyzed for *BdPMTprom::GUSPlus* associated GUS staining. The culms of plants at this juvenile age typically contained only one internode. GUS staining of sections throughout that internode was visible in the xylem vessels and tracheids (**Supplementary Figures S5A–C**), being similar to that observed in the apical internodes of 37-day-old plants (**Figure 2A**). Only occasionally was staining observed in sclerenchyma cells in the ring surrounding the vascular bundles and in the epidermal cell layer of the stem.

In 23-day-old *BdPMTprom::GUSPlus* plant stems as well as when using short X-Gluc solution incubation times on 37-day-old stem sections, indigo coloring was weak or not visible in cells constituting the phloem or in xylem parenchyma cells even though staining was clearly visible in xylem vessels and tracheids, suggesting that *BdPMT* promoter activity was relatively weaker in those cell types (**Supplementary Figures S5B,C**).

In *BdPMTprom::GUSPlus* T_1_- and T_2_-generation plants’ leaves, production of colorimetric product was strongest in major leaf veins, at the leaf margins, and in the leaf tip (**Figures 3A,F,G**; **Supplementary Figure S6**). Strong staining was also apparent in trichome macrohairs, whereas lighter staining occurred in developing and mature bicellular microhairs and in prickle hair cells at the leaf margin (**Figures 3F,G**; **Supplementary Figure S6**). Information on the anatomy and developmental control of trichome macrohair, bicellular microhair, and prickle hair cell formation in maize leaves can be found in Moose et al. (2004). *BdPMT* expression in these trichome subtypes was not unexpected given that lignification has been documented in the walls of Arabidopsis trichomes (Marks et al., 2008). *BdPMT* promoter-driven *GUS* expression was generally not detected or was weak in non-trichome epidermal cells except at the leaf margin and in the guard cells of stomata, where staining was prominent (**Figures 3F,G**).

*GUSPlus* expression driven by the *BdPMT* promoter was also assayed in 3-day-old roots of seedlings that had been germinated on selective agar growth media in vertically oriented plates. In seedlings from all four transgenic lines, GUS staining was visible in the vasculature and weakly in the root cap (**Figures 4A,F**; **Supplementary Figures S9A–D**). In the strongest expressing lines (**Supplementary Figures S9B,C**), staining was obvious in the root hairs. More work must be done to determine if root hair staining represents legitimate expression of the *BdPMT* gene or is instead an artifact related to transgene insertion site positional effects. Overall, staining was stronger in developmentally older tissues proximal to the seedling shoot compared to younger tissues closer to the root tip. This suggests that *BdPMT* promoter-driven expression of *GUSPlus* begins in developmentally young cells and continues into the mature zone. The above-described distinct staining patterns observed in the various plant organs differed from the uniform and ubiquitous staining patterns observed throughout plants transgenic for *ZmUBIQUITIN1prom* (*ZmUBI1prom*)*::GUSPlus* (**Figures 3D** and **4D**).

Microscopic examination of developing florets removed from the spikelets of 37-day-old plants and stained overnight with X-Gluc revealed strong GUS staining in the lemma, often to the degree that indigo precipitate became deposited in the solution between trichomes (**Figure 5**). Lesser staining was observed in the palea. The lemma and palea were carefully dissected away, and the internal floral organs removed and photographed. In these tissues, *BdPMTprom::GUSPlus*-related GUS staining was visible at the base of the ovary, in the feathery stigma, and lightly in pollen grains (**Figure 5E**; **Supplementary Figures S10A,B**).

### *BdCESA7* and *BdCESA8prom::GUSPlus* Expression Patterns were Indistinguishable from Each Other, but Varied Between Independent Lines

Eight independent lines each of *BdCESA7* and *BdCESA8prom::GUSPlus* T_1_ and T_2_-generation plants were analyzed for GUS staining in various tissues at different developmental stages (**Figures 1**–**5**; **Supplementary Figures S4**, **S5**, and **S7**–**S10**). Overall, the GUS staining patterns observed in plants harboring the *BdCESA7* promoter construct were indistinguishable from those of the *BdCESA8* promoter construct when comparing the same tissues at the same developmental stages. However, GUS staining patterns and intensities looked considerably different from line to line (**Supplementary Figures S4** and **S7**–**S9**).

In stem sections of flowering plants, GUS staining was consistently observed from line to line in xylem vessels and tracheids of vascular bundle cells, in interfascicular tissue sclerenchyma cells, and in epidermal cells (**Figures 1** and **2**); **Supplementary Figures S4** and **S5**). By contrast, GUS staining of cells in the cortex and in parenchyma cells was highly variable. In the strongest expressing *BdCESA7* and *BdCESA8prom::GUSPlus* lines, GUS staining in cortex cells in the youngest stem internodes was nearly as dark as that observed in vascular bundle cells, with dark staining extending throughout the entire stem sections including in the sclerenchyma and parenchyma cells (**Figures 1** and **2C,D**). Staining in bundle sheath cells and in phloem and xylary parenchyma cells, while visible in the strongest expressing lines, was faint or not visible in moderately and weakly expressing lines. This result is in line with the relatively weak signal detected in these tissues by *in situ hybridization* using a *BdCESA7* antisense probe, as reported in Handakumbura et al. (2013).

As with *BdPMTprom::GUSPlus* stem sections, *BdCESA7* and *BdCESA8prom::GUSPlus*-related GUS staining was strongest at the base of each internode where tissues were developmentally the youngest and were actively forming secondary cell walls (**Figures 1** and **2D–F**). Overall, staining appeared more ubiquitous in *BdCESA7* and *BdCESA8prom::GUSPlus* stem sections in comparison to that observed in *BdPMTprom::GUSPlus* plants’ stem sections of the same ages grown side by side. It cannot be ruled out, however, that if a larger number of *BdPMTprom::GUSPlus* independent transgenic lines were generated and analyzed, some might stain with comparably high intensity, given the observed large variability in expression levels likely caused by transgene chromosomal insertion site positional effects.

As with the stem sections, obvious line-to-line differences in the intensities of staining were observed in leaves of T_1_-generation *BdCESA7* and *BdCESA8prom::GUSPlus* plants (**Supplementary Figures S7** and **S8**). In all lines, *GUS* staining was detected in leaf veins, macrohair trichomes, and microhair trichomes (**Figures 3B,C**). In moderately expressing lines, GUS staining was patchy in leaf epidermal cells, whereas it was dark and uniform in highly expressing lines (**Supplementary Figures S7** and **S8**). By contrast, uniform staining was consistently observed in cells of *ZmUBIprom::GUSPlus* leaves from multiple lines (**Figure 3D**). *GUSPlus* expression driven by the *BdCESA7* and *BdCESA8* promoters was also assayed in 3 day-old roots, revealing GUS staining patterns similar to those observed in *BdPMTprom::GUSPlus* roots (**Figure 4**; **Supplementary Figure S9**). Equivalent GUS staining associated with the three different constructs was also observed in floral organs (**Figure 5**; **Supplementary Figure S10**).

### A *BdPMTprom::BdPMT ORF* Construct Rescued the *Bdpmt-1* Mutant Phenotype

**FIGURE 6:**
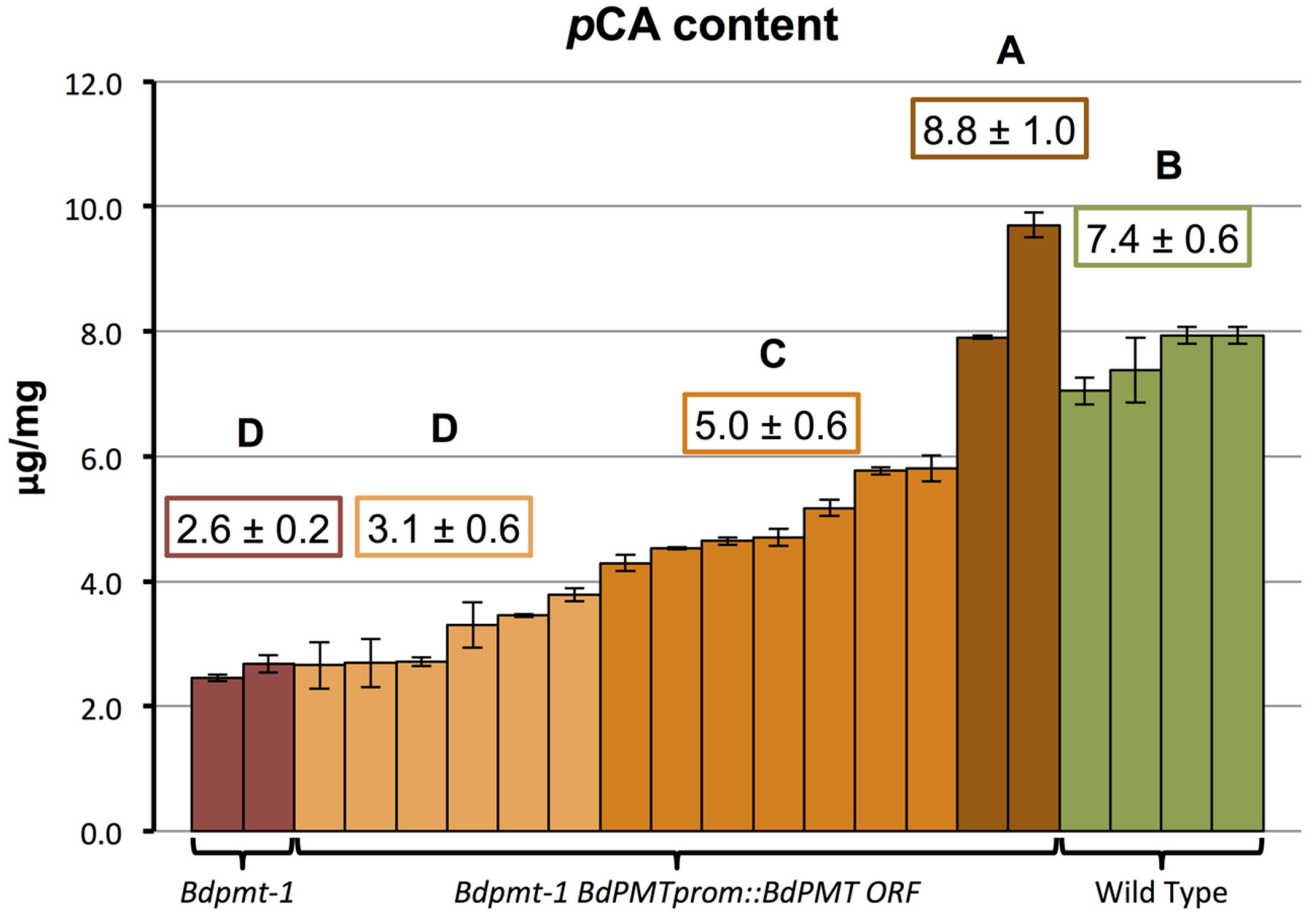
**Quantification of *p*-coumaric acid (*p*CA) released by 2 M NaOH from senesced stems of the following plants: *Bdpmt-1*; T_0_-generation *Bdpmt-1* harboring *BdPMTprom::BdPMT ORF* (15 independent events grouped into three statistically different groups where light orange = <50% rescue, dark orange (50—75% rescue, and brown (100% or greater rescue).** Means for different groups are shown in color-coded boxes. Letters represent statistically different groups. Bars represent standard errors of three technical replicates.

To validate that the *BdPMT* utility promoter vector was fully functional, and that the observed *BdPMTprom::GUSPlus* related GUS staining pattern likely reflected the expression pattern of the *BdPMT* gene, the *BdPMT* ORF was Gateway-cloned into the *BdPMT* utility promoter binary vector then transformed into the *Bdpmt-1* knockout mutant previously described in Petrik et al. (2014). A mild alkaline hydrolysis assay was performed on senesced stem tissue from 15 independent T_0_-generation *Bdpmt-1 BdPMTprom::BdPMT ORF* plants in order to determine if the construct rescued the reduced cell wall *p*CA phenotype. In *Bdpmt-1* mutant senesced stems, cell wall *p*CA levels were measured to be about 35% that of WT stems (**Figure 6**). Senesced stems from the 15 *Bdpmt-1 BdPMTprom::BdPMT ORF* independent transformants were found to contain a range of cell wall *p*CA (**Figure 6**). Two of the transformants had *p*CA levels higher than that of WT, whereas *p*CA content in seven transformants was intermediate between WT and the *Bdpmt-1* mutant stems. *p*CA content in the remaining six transformants were statistically indistinguishable from that in the *Bdpmt-1* mutant stems. These results suggest that the *BdPMTprom::BdPMT ORF* construct is functional, but that chromosome insertion site effects greatly affected expression levels such that most lines did not have full rescue of the *Bdpmt-1* mutant phenotype.

### A *Bdcesa8* T-DNA Insertional Mutant Exhibited Substantially Reduced Crystalline Cellulose and Stunted Culms that Could be Rescued by a *BdCESA8prom::BdCESA8 ORF* Construct

**FIGURE 7:**
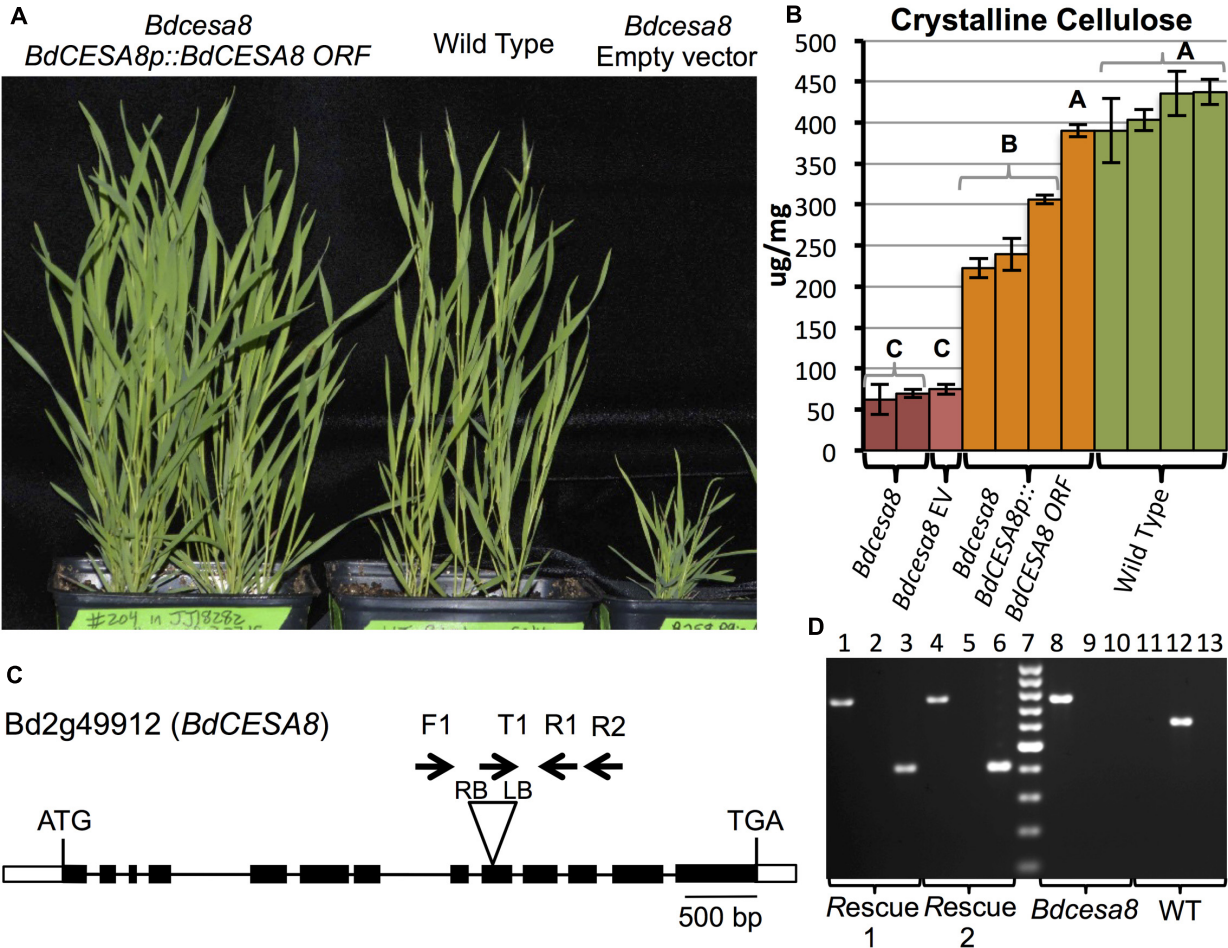
**Transgenic rescue of the *Bdcesa8-1* stunted culm growth and reduced cellulose phenotypes.** **(A)** T_1_-generation homozygous *Bdcesa8-1* plants harboring the *BdCESA8prom::BdCESA8 ORF* construct (left pot) exhibit culm growth comparable to WT plants (center pot). Right pot contains a homozygous *Bdcesa8-1* plant transformed with the empty vector (no rescue). **(B)** Crystalline cellulose content of senesced stems from the following plants: *Bdcesa8-1*; *Bdcesa8-1* containing the empty vector (*Bdcesa8* EV); *Bdcesa8-1* containing *BdCESA8prom::BdCESA8 ORF*; Wild type. Bars represent standard errors, three technical replicates. Different letters represent statistical differences. **(C)** Diagram of the *BdCESA8* gene Bd2g49912 drawn to scale. Bars and lines represent exons and introns, respectively. Shown is the location of the T-DNA insertion (triangle) in the *Bdcesa8-1* mutant line JJ18282 along with relative primer locations (arrows). RB and LB represent T-DNA right border and left border. **(D)** Agarose gel-electrophoresed PCR products, scoring for the presence of the *Bdcesa8-1* T-DNA (lanes 1, 4, 8, 11; primers Bdcesa8-1_T-DNA_LB_T1 (T1) + BdCESA8_EXON11_R2 (R2), 756 bp product) or the absence of the T-DNA (lanes 2, 5, 9, 12; primers BdCESA8_INTRON7_F1 (F1) + BdCESA8_EXON10_R1 (R1), 627 bp product) as well as the presence or absence of the *BdCESA8prom::BdCESA8 ORF* rescue construct (lanes 3, 6, 10, 13; primers BdCESA8_EXON13_F + NOST_R, 408 bp product). Rescue 1 and 2 are two independent *Bdcesa8-1* plants transformed with *BdCESA8prom::BdCESA8 ORF* (plants shown in **A**). Lane 7 is the Fermentas GeneRuler 100 bp ladder (cat #SM0241).

To validate that the *BdCESA8* promoter fragment contained the regulatory sequences necessary and sufficient to drive expression of the *BdCESA8* ORF to rescue the phenotype of a *Bdcesa8* mutant, we first had to identify a *Bdcesa8* mutant and confirm it had a phenotype consistent with *BdCESA8* involvement in secondary cell wall cellulose deposition. A query of the Brachypodium mutant collection database identified, a T-DNA line, JJ18282, with an insertion in the 9th exon of the 13-exon *BdCESA8* (Bradi2g49912) (**Figure 7**). PCR primers were designed to score the presence or absence of the T-DNA so as to identify homozygous and heterozygous plants (**Figures 7C,D**; **Supplementary Table S1**). Homozygous *Bdcesa8-1* plants were found to exhibit stunted culm growth (**Figure 7A**; **Supplementary Figures S12A**) along with a sterile phenotype and spikelets with awns that became distorted upon maturation (**Supplementary Figure S12B**). Crystalline cellulose levels in homozygous *Bdcesa8-1* senesced stems were 16 to 26% that of WT stems (**Figure 7B**; **Supplementary Figure S12C**). Given that culm growth and cellulose content of heterozygous *BdCESA8*/*Bdcesa8-1* plants were indistinguishable from WT, the *Bdcesa8-1* mutation is considered to be recessive.

Since the T-DNA insertion present in the *Bdcesa8-1* mutant harbored the *HPT* selectable marker gene, the *BdCESA8* utility promoter binary vector could not be employed to test rescue of the *Bdcesa8-1* mutant phenotype given that it also contained the *HPT* gene. Therefore, the *BdCESA8* promoter cassette containing the *BdCESA8* ORF was cloned into the B258 p9ioACT binary vector, which employs the *NPTII* selectable marker (**Supplementary Figure S11**), and introduced into *Bdcesa8-1* plants. Nine T_0_-generation plants homozygous for *Bdcesa8-1* and harboring independent insertions of the *BdCESA8prom::BdCESA8* ORF construct were identified and confirmed by PCR (**Figure 7D**). Of those plants, seven had culm growth similar to that of WT along with partial to full seed set (**Figure 7A**). Analysis of crystalline cellulose content in senesced stems from four of the independent transformants, that all had normal culm height, revealed that one transformant had cellulose content indistinguishable from that of WT whereas the other three transformants had cellulose contents intermediate between those of homozygous *Bdcesa8-1* plants and WT (**Figure 7B**). Therefore, as observed with the *BdPMTprom::BdPMT* ORF-containing *Bdpmt-1* plants, only a fraction of the *BdCESA8prom::BdCESA8* ORF-containing *Bdcesa8-1* plants exhibited full phenotypic rescue.

## Discussion

In this study, we showed that *Brachypodium* plants stably transformed with either the *BdPMTprom::GUSPlus*, *BdCESA7prom::GUSPlus*, or *BdCESA8prom::GUSPlus* construct exhibited GUS staining patterns that would be expected for genes involved in secondary cell wall biosynthesis. The functionality of the *BdPMT* and *BdCESA8* utility promoter binary vectors were validated by demonstrating that they could drive expression of the *BdPMT ORF* and *BdCESA8 ORF* to rescue the *Bdpmt-1* and *Bdcesa8-1* mutant phenotypes, respectively. The *Bdcesa8-1* T-DNA insertional mutant that we identified exhibited stunted culm growth and drastically reduced crystalline cellulose levels in stem tissues, which is consistent with *BdCESA8* functioning as a cellulose synthase involved in secondary cell wall deposition.

Given that the expression patterns of the *BdCESA7* and *BdCESA8* promoters were indistinguishable, this combination of promoters will have utility in applications where two different genes are to be expressed with identical spatiotemporal patterns. Gene silencing can occur when the same promoter is used to drive expression of more than one gene (Park et al., 1996). The use of these two promoters should circumvent such an effect, given that the promoters share minimal DNA sequence identity.

Previously we determined that the *Brachypodium PMT* gene encodes a BAHD (BEAT, AHCT, HCBT, and DAT) acyltransferase that specifically acylates monolignols with *p*-coumarate (*p*CA) thereby producing monolignol *p*CA (ML-*p*CA) conjugates incorporated into lignin in secondary-cell-wall-forming tissues (Withers et al., 2012; Petrik et al., 2014). As such, the *BdPMT* promoter construct should have utility in driving gene-of-interest expression spatially and temporally in cells involved in lignin deposition.

Handakumbura et al. (2013) showed that the *BdCESA4* (Bradi3g28350) and *BdCESA7* (Bradi4g30540) genes are involved in cellulose biosynthesis in secondary cell wall-forming tissues of *Brachypodium*. In addition, they found that *BdCESA8* (Bradi2g49912) exhibited an expression pattern equivalent to *BdCESA4* and *BdCESA7*, suggesting that they may function together. In other plant species including *Oryza sativa* (rice), three *CESA* genes function together and non-redundantly in secondary cell wall cellulose biosynthesis (Tanaka et al., 2003; Hill et al., 2014). BdCESA8 is the closest *Brachypodium* homolog to the secondary-cell-wall-forming rice OsCESA4 cellulose synthase (Tanaka et al., 2003; see phylogenetic tree in Handakumbura et al., 2013). Moreover, Valdivia et al. (2013) found that *BdCESA8* was strongly upregulated when the NAC transcription factor *BdSWN5*, which controls secondary cell wall biosynthesis, was overexpressed in *Brachypodium*.

The observed *Bdcesa8-1* phenotypes including the reduced fertility phenotype are equivalent to those associated with loss-of function mutations in the rice secondary-cell-wall-forming *OsCESA4*, *OsCESA7*, and *OsCESA9* genes (Tanaka et al., 2003). Handakumbura et al. (2013) reported similar phenotypes in *Brachypodium* lines targeted for reduced *BdCESA4* or *BdCESA7* expression using artificial microRNAs, although those reported phenotypes were not as strong as observed in our *Bdcesa8-1* T-DNA insertional mutant, which is likely a null, presumably because the microRNAs conferred only partial loss of function.

Our results, taken together with the above-noted data, strongly suggest that *BdCESA8* is involved in the synthesis of cellulose in secondary cell walls. Loss of function of a primary cell wall cellulose synthase would not explain the greater than 80% reduction in *Bdcesa8-1* crystalline cellulose levels in senesced stem tissue, which is a tissue type that is mostly composed of secondary cell wall material. Moreover, we did not observe *Bdcesa8-1* mutant phenotypes that would be expected for a cellulose synthase involved in primary cell wall biosynthesis, which include severe cell expansion defects in embryos and seedlings (Beeckman et al., 2002; Persson et al., 2007).

Histochemical assessment of *Brachypodium* plants transgenic for the *BdPMT, BdCESA7*, and *BdCESA8prom::GUSPlus* constructs showed expression predominantly in xylem vessels, tracheids, and interfascicular fiber cells in stem sections as well as in vasculature of leaves and roots (**Figures 1–4**). Although expression patterns were similar between independent transgenic plants for each promoter tested, the strength of expression varied considerably between the transgenic lines (**Supplementary Figures S8**–**S12**). For example, of the eight independent *BdCESA8prom::GUSPlus* lines, one line had a high GUS expression level, four lines had medium GUS expression, and three lines had low GUS expression levels (**Supplementary Figure S8**). These differences were likely due, at least in part, to the effects of *cis-* and *trans*-regulatory elements in the surrounding native genomic DNA proximal to the integrated T-DNA insertion sites (Xue et al., 2005; Ziemienowicz, 2010). Differences in transgene copy number could also underlie the expression level differences. In that regard, the *Agrobacterium*-mediated transformation method used in this study can result in more than one T-DNA insertion per plant line (1.5 insertions on average; Bragg et al., 2012).

Gene expression differences between transgenic lines may be desirable in instances where complete loss of function or too-high overexpression causes severe phenotypes or lethality. Efforts to mediate transgene expression variation across transgenic lines have included flanking the transgene with scaffold/matrix attachment regions (S/MARs; Breyne et al., 1992; Xue et al., 2005). However, although S/MAR inclusion often increased transgene expression levels overall, variation across transgenic lines were reported to still occur (Nowak et al., 2001).

*BdPMT, BdCESA7*, and *BdCESA8prom::GUSPlus*-derived GUS staining was also observed in floral tissues at the base of the ovary, in the feathery style, and within pollen grains (**Figure 5**; **Supplementary Figure S10**). This finding could indicate that the *BdCESA7* and *BdCESA8* genes are involved in cellulose biosynthesis within floral organs and within pollen grain walls (Ferguson et al., 1998), which is consistent with the fact that loss of *BdCESA8* function resulted in sterility. *BdPMT* expression in pollen may be related to biosynthesis of phenolics deposited in the intine of the pollen grain (Quilichini et al., 2015). Grienenberger et al. (2009) demonstrated that a BAHD acyltransferase distantly related to *BdPMT* was responsible for hydroxycinnamoyl spermidine biosynthesis in the tapetum layer of the *Arabidopsis thaliana* anther, with the synthesized compounds becoming deposited in the pollen grain exine. It is worth noting that no pollen viability phenotype was observed in the *Bdpmt-1* null mutant.

Although vascular-specific promoters have been isolated from and effectively used in dicot plant species (see Introduction), dicot-derived promoters do not always work efficiently in monocot species (Schledzewski and Mendel, 1994). To date, only a few grass-species-derived vascular-specific promoters have been cloned and characterized. These include promoters of the rice *OsHOX1*, *OsSWN*, and *OsCESA9* genes. The *OsHOX1* promoter fragment conferred vascular-specific *GUS* expression that was modulated by sucrose and auxin (Scarpella et al., 2000). It’s expression pattern differed from those characterized in this study in that it was more specific to the vasculature. Vascular-specific GUS expression was also observed for the *OsSWN2* promoter fragment (Yoshida et al., 2013). By contrast, the *OsSWN1* promoter fragment conferred GUS expression in rice stem sections more similar to that observed in this study, with GUS staining observed in several layers of sclerenchymatous cells beneath the epidermis as well as in the vasculature (Yoshida et al., 2013).

The *OsCESA9* promoter fragment also drove GUS expression in rice culms differently from that reported here for the *BdCESA7* and *BdCESA8* promoter fragments. *OsCESA9* and *BdCESA7* are orthologs. Kotake et al. (2011) observed *OsCESA9prom::GUS* staining only in the vasculature and not in sclerenchymatous cells as one would expect, given *in situ* hybridization data (Li et al., 2003; Handakumbura et al., 2013) and the fact that mutations in *OsCESA9* result in reduced cell wall thickness in sclerenchymatous cells (Kotake et al., 2011). It may be that the *OsCESA9* promoter fragment was missing important regulatory elements or that chromosomal insertion site positional effects influenced the expression pattern of the construct.

## Conclusion

It is anticipated that the utility promoter binary vector constructs characterized in this study could be used to drive gene-of-interest expression in secondary-cell-wall-forming tissues of various grass species such as switchgrass, wheat, rice, or maize, given that other monocot promoter sequences have been shown to be functional in related species, including Brachypodium-derived promoters in maize (Coussens et al., 2012; Thilmony et al., 2014). These utility vectors should hold value in application in model species such as *Brachypodium distachyon* as well as in monocot crops of commercial interest.

## Author Contributions

DP, JV, JR, and JS conceived of the experiments and interpreted data. DP, CC, SK, CF, and DP carried out experiments and analyzed data. DP, CC, and JS wrote the manuscript. All authors edited and approved the final manuscript.

## Conflict of Interest Statement

The authors declare that the research was conducted in the absence of any commercial or financial relationships that could be construed as a potential conflict of interest.

## Footnotes

1 http://aranet.mpimp-golm.mpg.de/

2 www.PlantGDB.org

3 phytozome.jgi.doe.gov/

4 http://www.dna-cloning.com/

5 http://jgi.doe.gov/our-science/science-programs/plant-genomics/brachypodium/brachypodium-t-dna-collection/.
