# Supplementary material for: BdCESA7, BdCESA8, and BdPMT Utility Promoter Constructs for Targeted Expression to Secondary Cell-Wall-Forming Cells of Grasses
Source: Front Plant Sci. 2016 Feb 4;7:55. doi: 10.3389/fpls.2016.00055 (PMC4740387; doi:10.3389/fpls.2016.00055)
Supplement: Supplementary file 1 [file Presentation_1.PDF]

SUPPLEMENTARY DATA

BdCESA7, *BdCESA8*, and *BdPMT* utility promoter constructs for targeted expression to secondary cell-wall-forming cells of grasses

Deborah L. Petrik,[1,2] Cynthia L. Cass,[1,2] Dharshana Padmakshan,[2] Cliff E. Foster,[3] John P. Vogel,[4] Steven D. Karlen,[2] John Ralph,[2,5] and John C. Sedbrook[1,2*]

[1]School of Biological Sciences, Illinois State University, Normal, IL 61790, USA

[2]U.S. Department of Energy Great Lakes Bioenergy Research Center, University of Wisconsin, Madison, WI 53706, USA

[3]U.S. Department of Energy Great Lakes Bioenergy Research Center, Michigan State University, East Lansing, Michigan 48824, USA

[4]U.S. Department of Energy Joint Genome Institute, Walnut Creek, CA 94598, USA

[5]Department of Biochemistry, Wisconsin Energy Institute, University of Wisconsin, Madison, WI, 53706, USA

*Address for correspondence: John Sedbrook, School of Biological Sciences, Illinois State University, Normal, IL 61790, phone 309-438-3374, fax 309-438-3722, email jcsedbr@ilstu.edu

Supplemental Table 1. Primers used to amplify promoter sequences of *BdCESA7* Bradi4g30540, *BdCESA8* Bradi2g49912, *BdPMT* Bradi2g36910, and to test inheritance of *Bdcesa8-1* T-DNA insertion and *BdCESA8 ORF* phenotypic rescue.

Primer name	Sequence	Use
BdCESA7proSwaI_F	GAGAGATTTAAATTGTCGTGGTACGGAGGAGCTAG	Promoter cloning
BdCESA7proAscI_R	GAGAGGCGCGCCCGCCGGCCGGTCGATCTCCGC	Promoter cloning
BdCESA8proStuI_F	GAGAGAGGCCTGCTGCCACGCTGGTGAAGG	Promoter cloning
BdCESA8proAscI_R	GAGAGGCGCGCCGGCTCTCCCTCCGTGCG	Promoter cloning
BdPMTproSwaI_F	GAGAGATTTAAATGATGCGGAGGAGATAG	Promoter cloning
BdPMTproRAscI_R	GAGAGGCGCGCCTAAGCTTGCATGCTCTAGC	Promoter cloning
NOST_R	GCAAGACCGGCAACAGGATT	Genotyping
BdCESA8_EXON13_F	CCTGGTGCTCAACCTTGTCG	Genotyping
Bdcesa8-1 T-DNA LB T1	AGCTGTTTCCTGTGTGAAATTG	Genotyping
BdCESA8_EXON11_R2	GAAGACTGCCCAAACGACTT	Genotyping
BdCESA8_INTRON7_F1	TTGATGCTTGCTGATCCTTCCTAG	Genotyping
BdCESA8_EXON10_R1	CTGGTCCTTGGATGCCATCAAGC	Genotyping
ZmUbiIN1AscI_F	GAGAGGCGCGCCGTACGCCGCTCGTCCTCC	Cloning
ZmUbiIN1HindIII_R	GAGAAAGCTTCTGCAGAAGTAACACC	Cloning
GUSPLUS_BamHI_F	GAGAGGATCCATGGTAGATCTGAGGGT	Cloning
GUSPLUS_XhoI_R	GAGACTCGAGTCACACGTGATGGTGATGGTG	Cloning
BdPMTBamHI_F	GAGAGGATCCGATGGAGAAGAAGTTCACGGTG	ORF cloning
BdPMTSpeIXhoI_R	GAGAACTAGTCTCGAGTCACTTCCCGGCGGTGAAG GCGAAGGCG	ORF cloning
BdCESA8SalI_F	GAGAGTCGACGATGATGGAGTCGGGGACCCATC	ORF cloning
BdCESA8XhoI_R	GAGACTCGAGTCAGCAGTCGATGGAGCTGC	ORF cloning

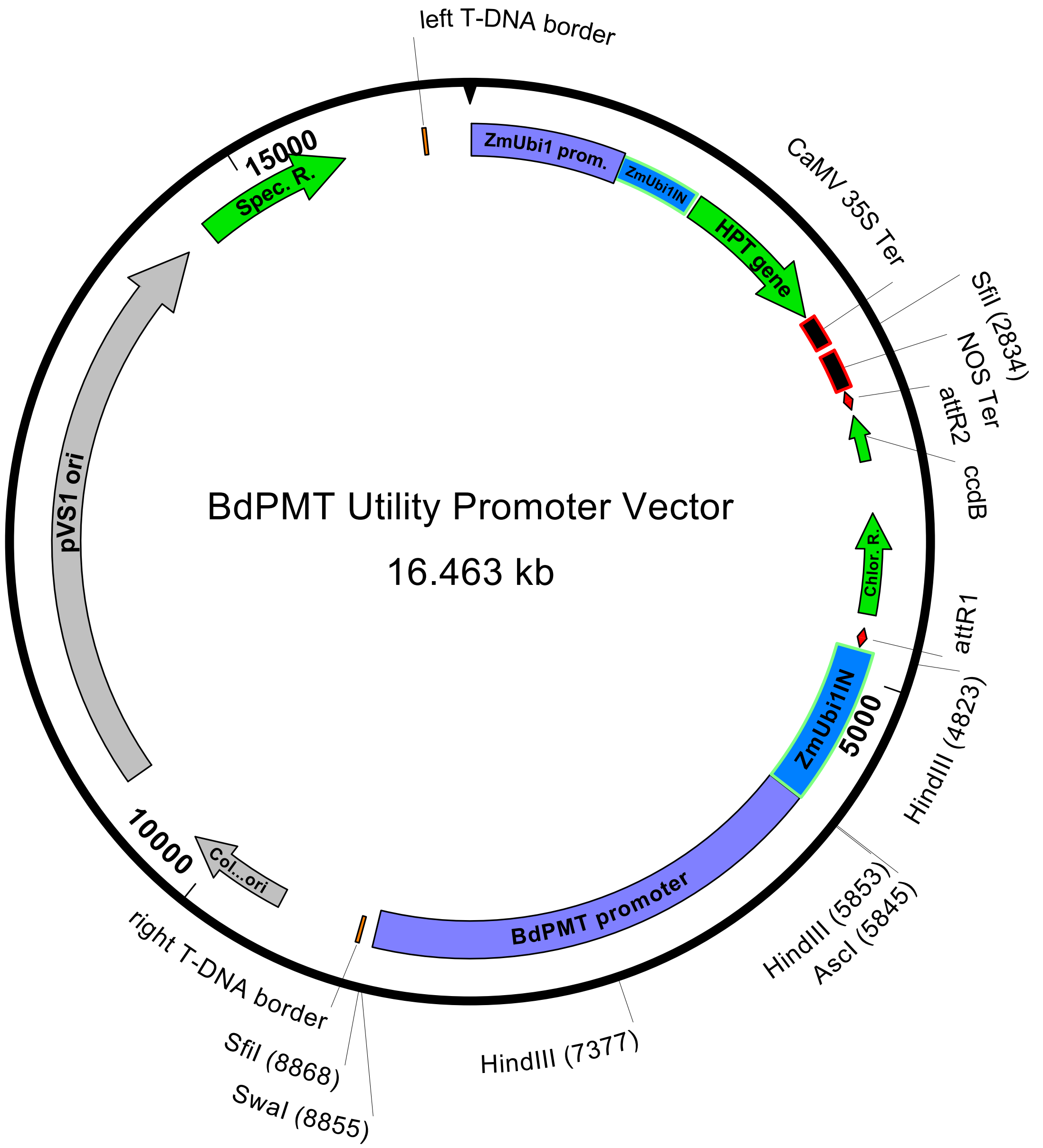

Supplemental Figure 1. Map of the *BdPMT* utility promoter binary vector. The *BdPMT* (*Bradi2g36910*) gene promoter plus the *Zea mays UBIQUITIN1* intron (*ZmUbi1IN*) were placed upstream of a Gateway-cloning compatible cassette to make the binary construct. Selection is Hygromycin B in plants and Spectinomycin in bacteria. Abbreviations are as follows: Chlor. R. = Chloramphenicol resistance gene; ccdB = DNA Gyrase "kill" gene; NOS ter = Nopaline synthase terminator; CaMV 35S Ter = Cauliflower mosaic virus 35S terminator; HPT gene = *HYGROMYCIN PHOSPHOTRANSFERASE II*. Note that the *ZmUbi1IN* adjacent to the *ZmUbi1* promoter is a truncated version of that adjacent to the *BdPMT* promoter.

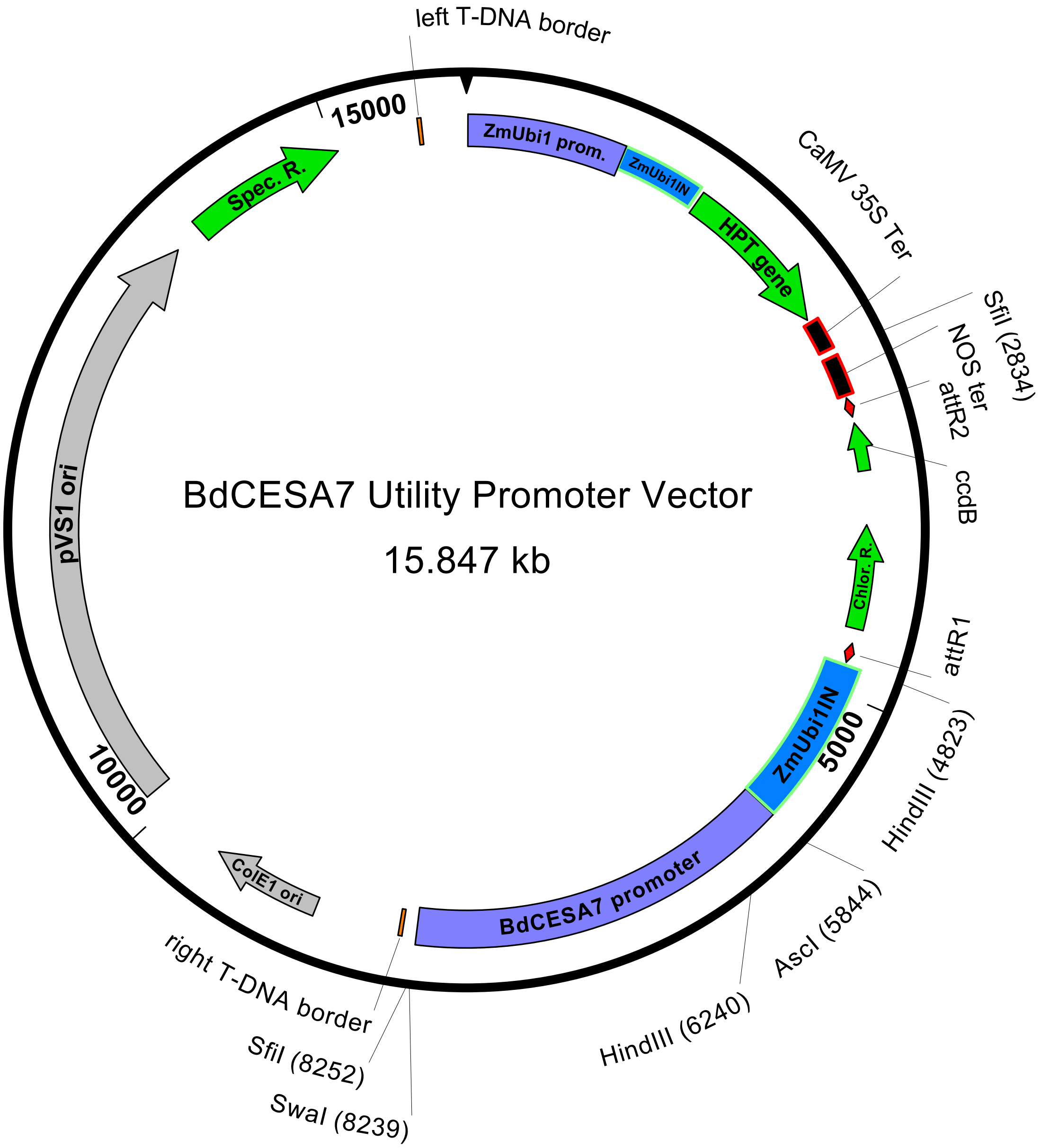

Supplemental Figure 2. Map of the *BdCESA7* utility promoter binary vector. See Supplemental Figure 1 legend for definitions of abbreviations.

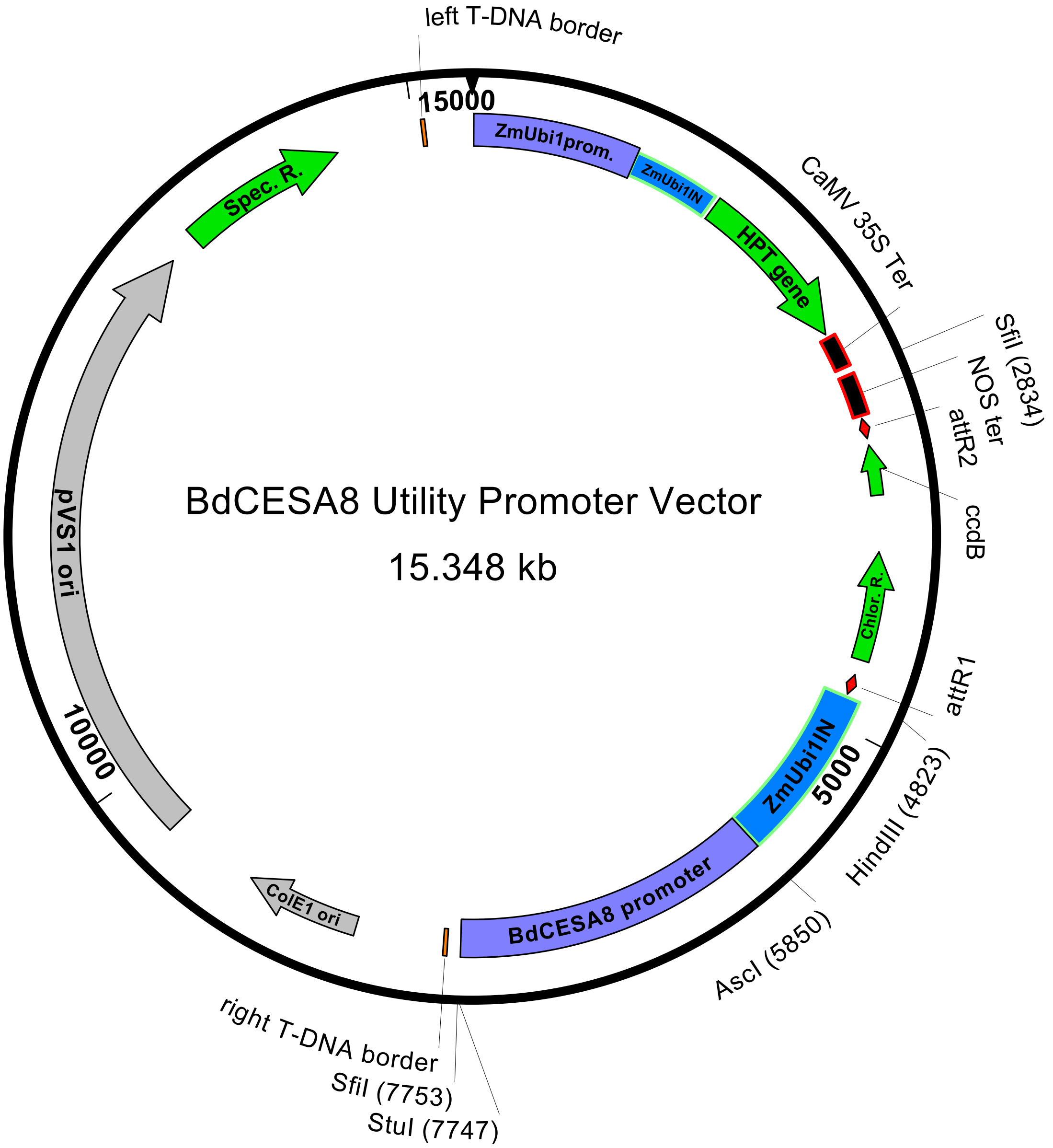

Supplemental Figure 3. Map of the *BdCESA8* utility promoter binary vector. See Supplemental Figure 1 legend for definitions of abbreviations.

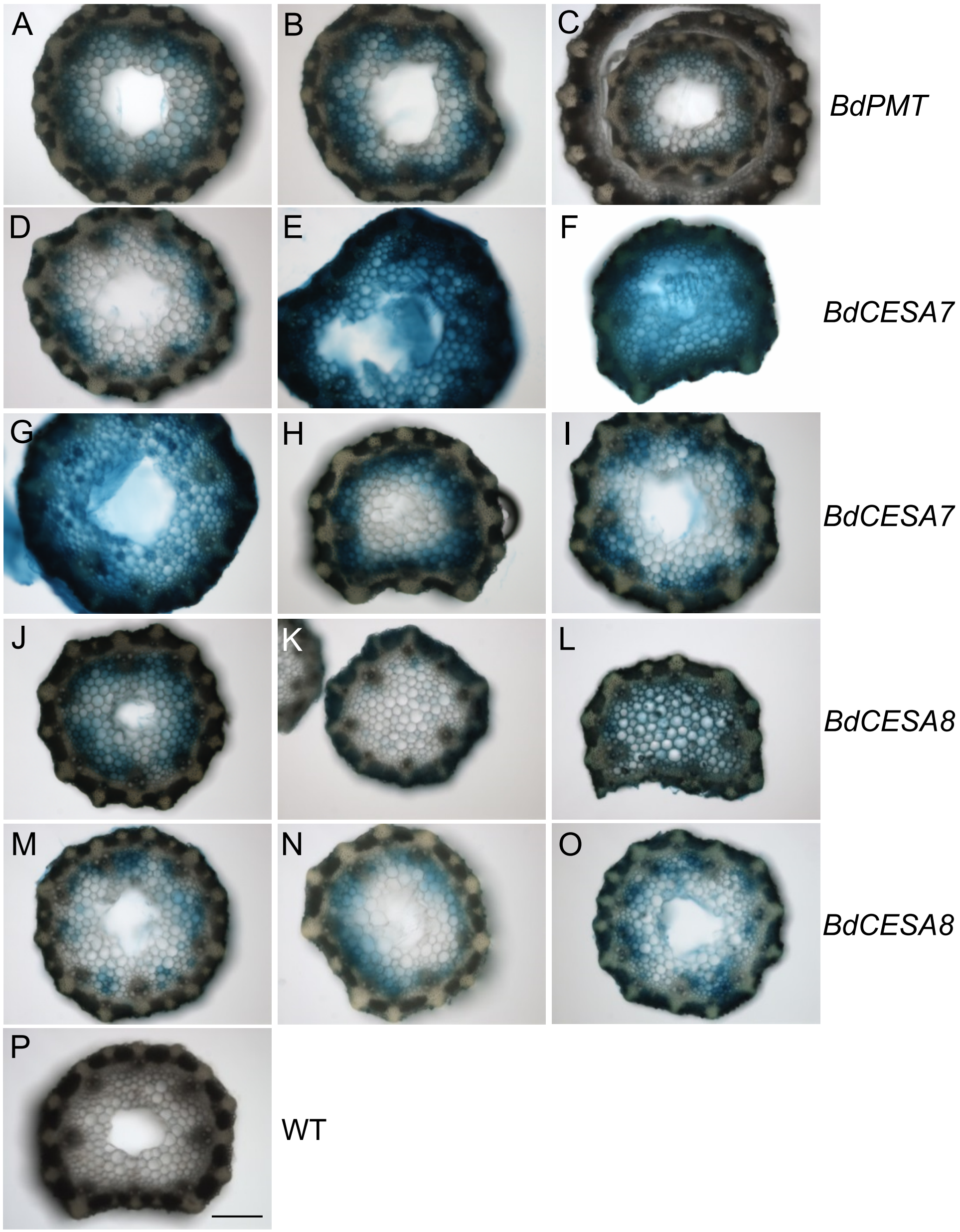

Supplemental Figure 4. Comparison of GUS expression in apical internode cross sections taken from independent transgenic lines, 37-day-old plants. All sections were stained the same duration. **A-C)** *BdPMTprom::GUSPlus.* **D-I)** *BdCESA7prom::GUSPlus*. **J-O)** *BdCESA8prom::GUSPlus*. **P)** Wild type. Scale bar = 170 µm.

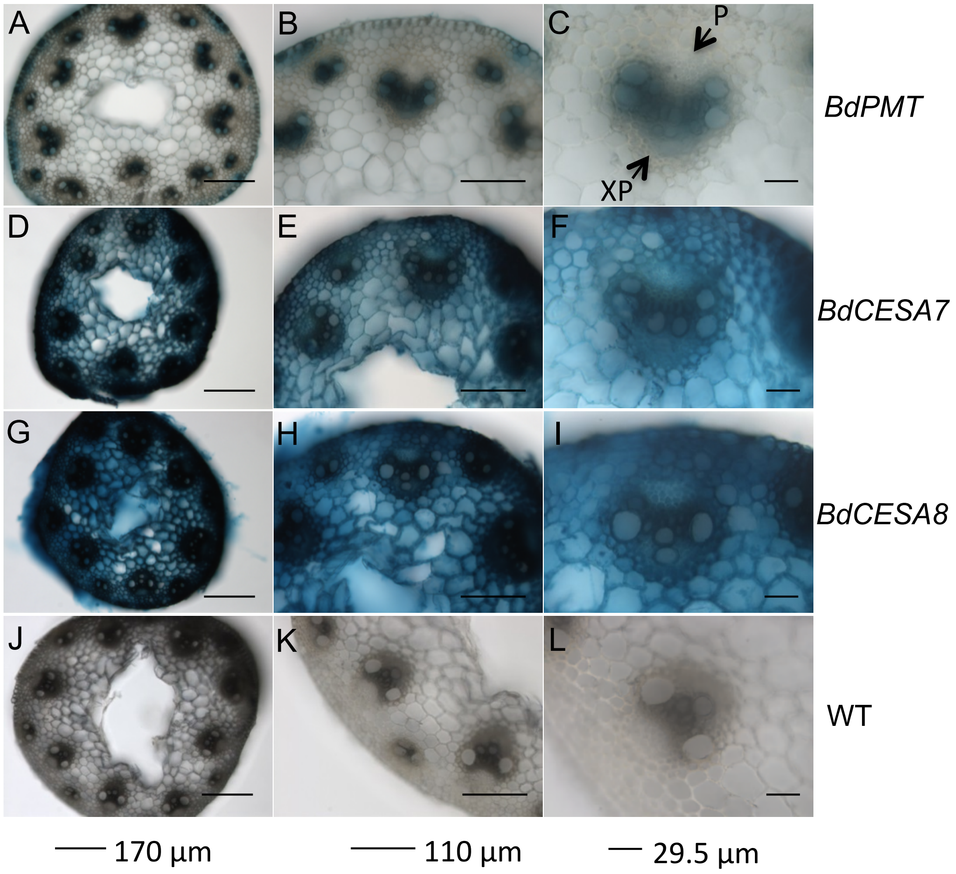

Supplemental Figure 5. GUS-stained stem cross sections, taken near the base of apical internode, from 23-day-old plants transformed with **A-C)** *BdPMTprom::GUSPlus*; **D-F)** *BdCESA7prom::GUSPlus*; **G-I)** *BdCESA8prom::GUSPlus*; **J-L)** Wild type. P = Phloem; XP = Xylem Perenchyma. Scale bar sizes are listed at the bottom.

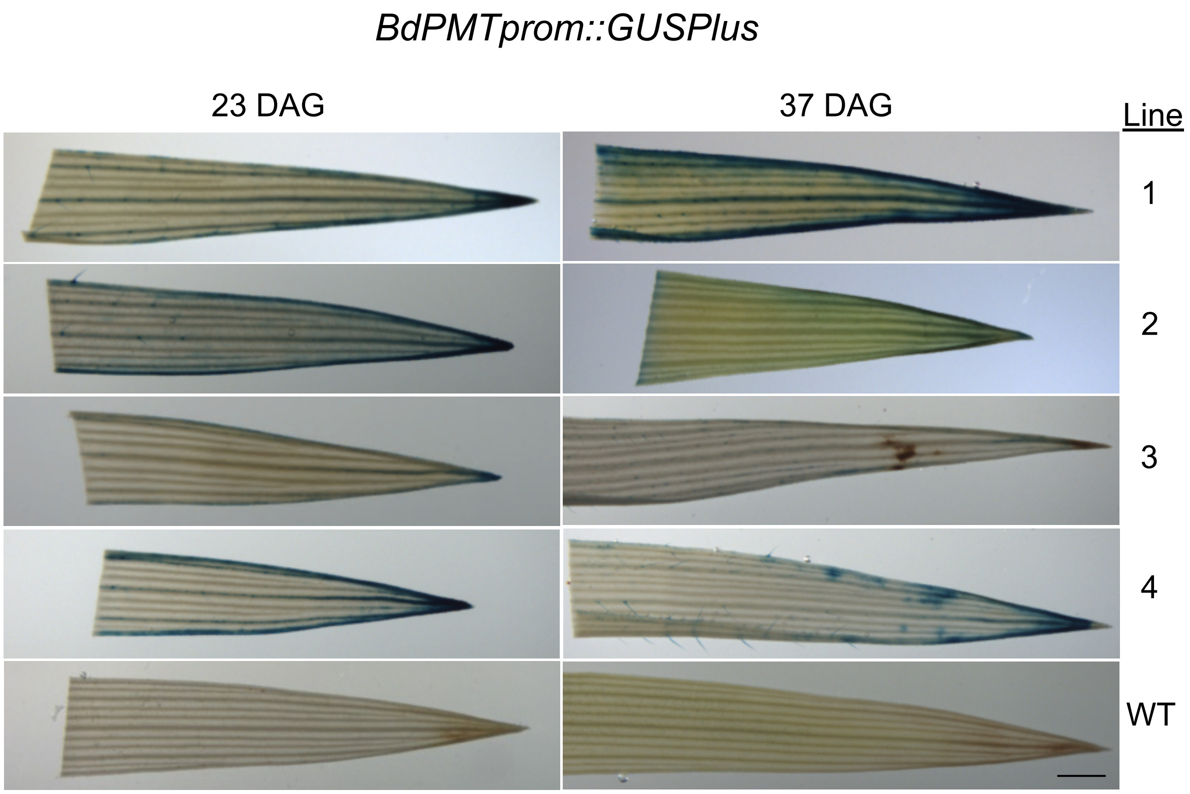

Supplemental Figure 6. Comparison of relative GUS expression in 23-day-old (left panels) and 37-day-old leaves (right panels) of four *BdPMTprom::GUSPlus* independent transgenic lines. DAG = days after germination. Scale bar = 1 mm.

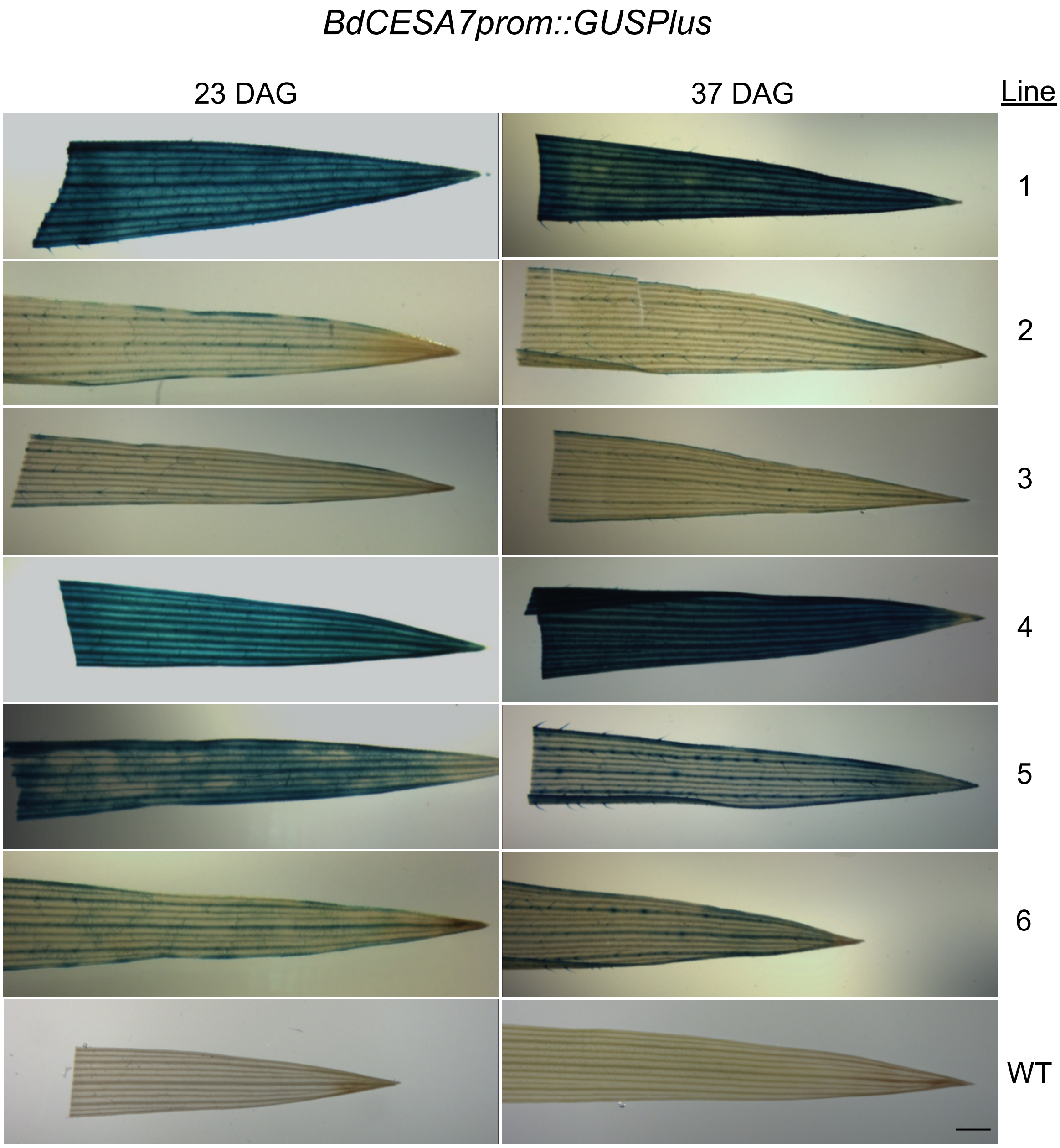

Supplemental Figure 7. Comparison of relative GUS expression in the leaves of six *BdCESA7prom::GUSPlus* independent transgenic lines. Left and right panels depict GUS-stained juvenile and adult leaves, respectively. DAG = days after germination. Scale bar = 1 mm.

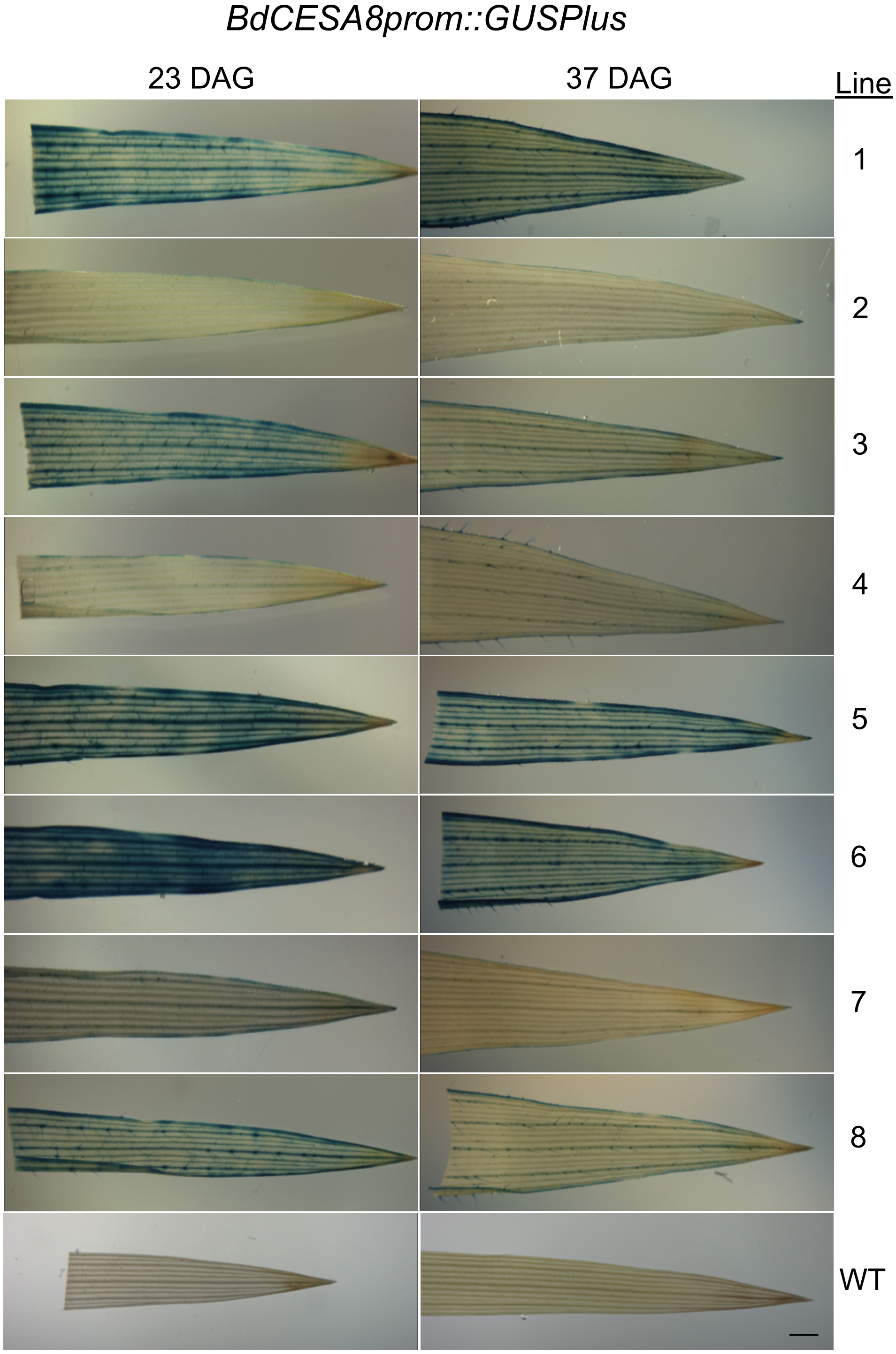

Supplemental Figure 8. Comparison of relative GUS expression in the leaves of eight *BdCESA8prom::GUSPlus* independent transgenic lines. Left and right panels depict GUS-stained juvenile and adult leaves, respectively. DAG = days after germination. Scale bar = 1 mm.

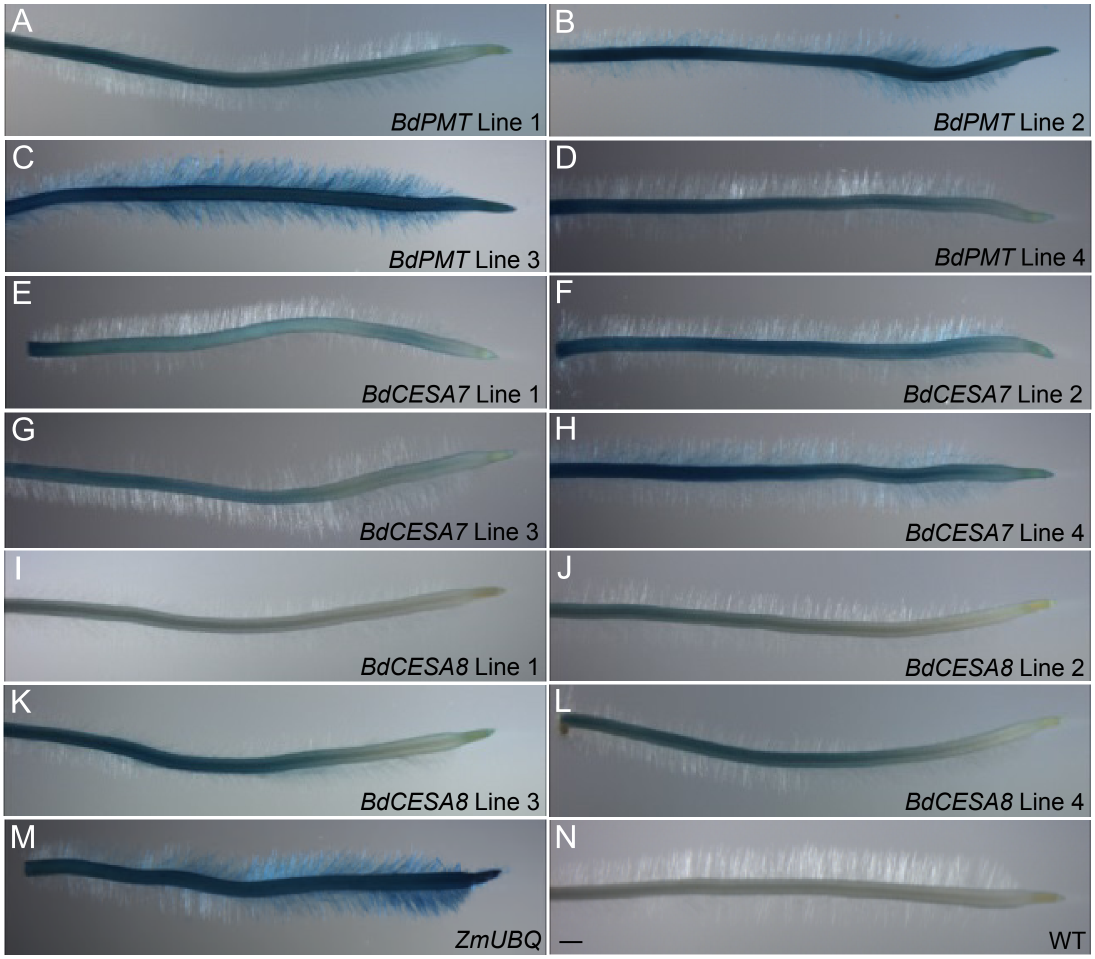

Supplemental Figure 9. Comparison of relative GUS expression in 3-day-old seedling roots from independent transgenic lines. **A-D)** *BdPMTprom::GUSPlus.* **E-H)** *BdCESA7prom::GUSPlus*. **I-L)** *BdCESA8prom::GUSPlus*. **M)** *ZmUBQprom::GUSPlus*. **N)** Wild type. Scale bar = 1 mm.

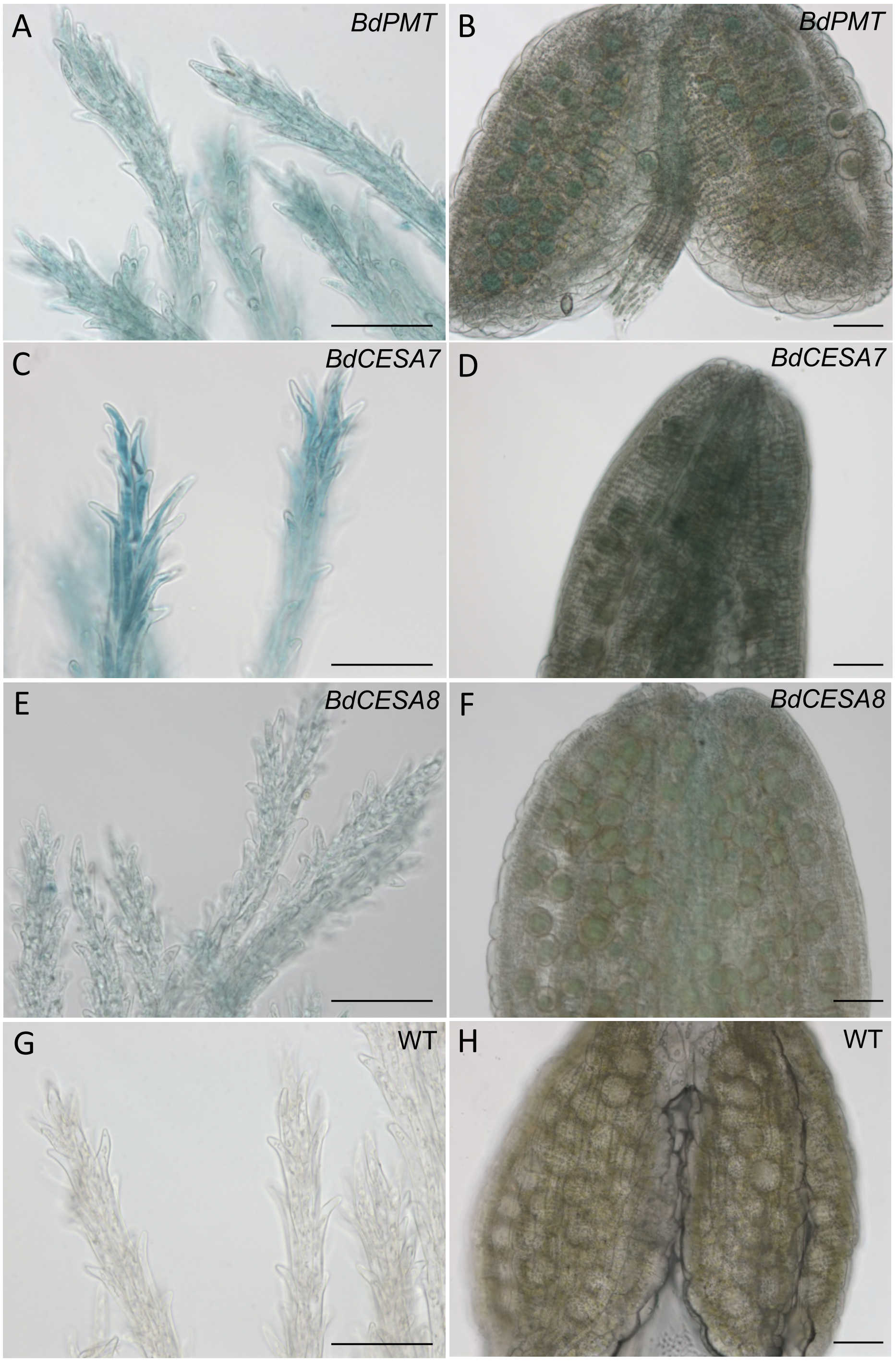

Supplemental Figure 10. GUS-stained stigmas and anthers. **A, B)** *BdPMTprom::GUSPlus.* **C, D)** *BdCESA7prom::GUSPlus*. **E, F)** *BdCESA8prom::GUSPlus*. **G, H)** Wild type. Note the GUS staining of pollen in **B**, **D**, and **F**. Scale bars = 50 μm.

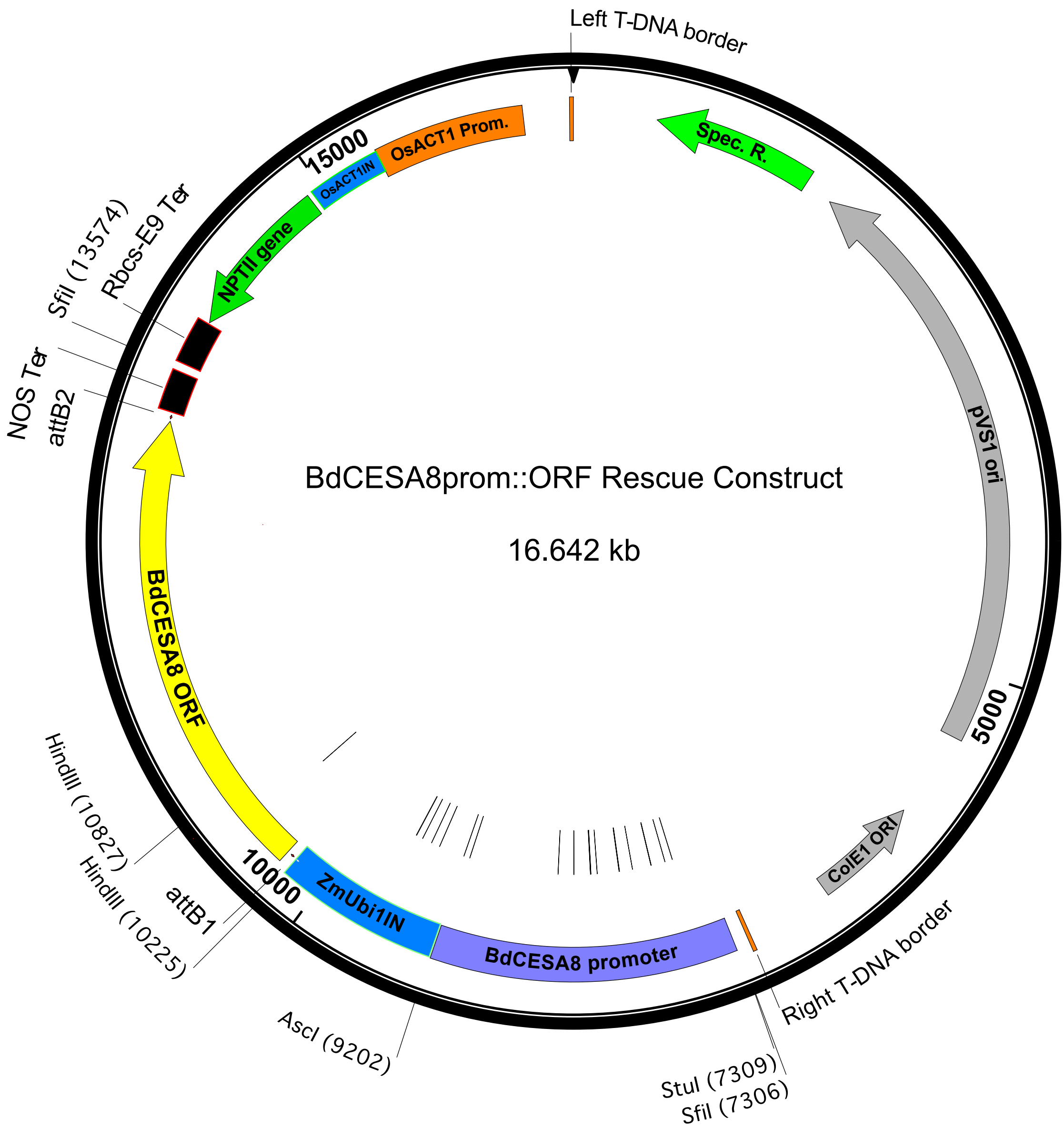

Supplemental Figure 11. Map of the *BdCESA8prom::BdCESA8 ORF* rescue construct.

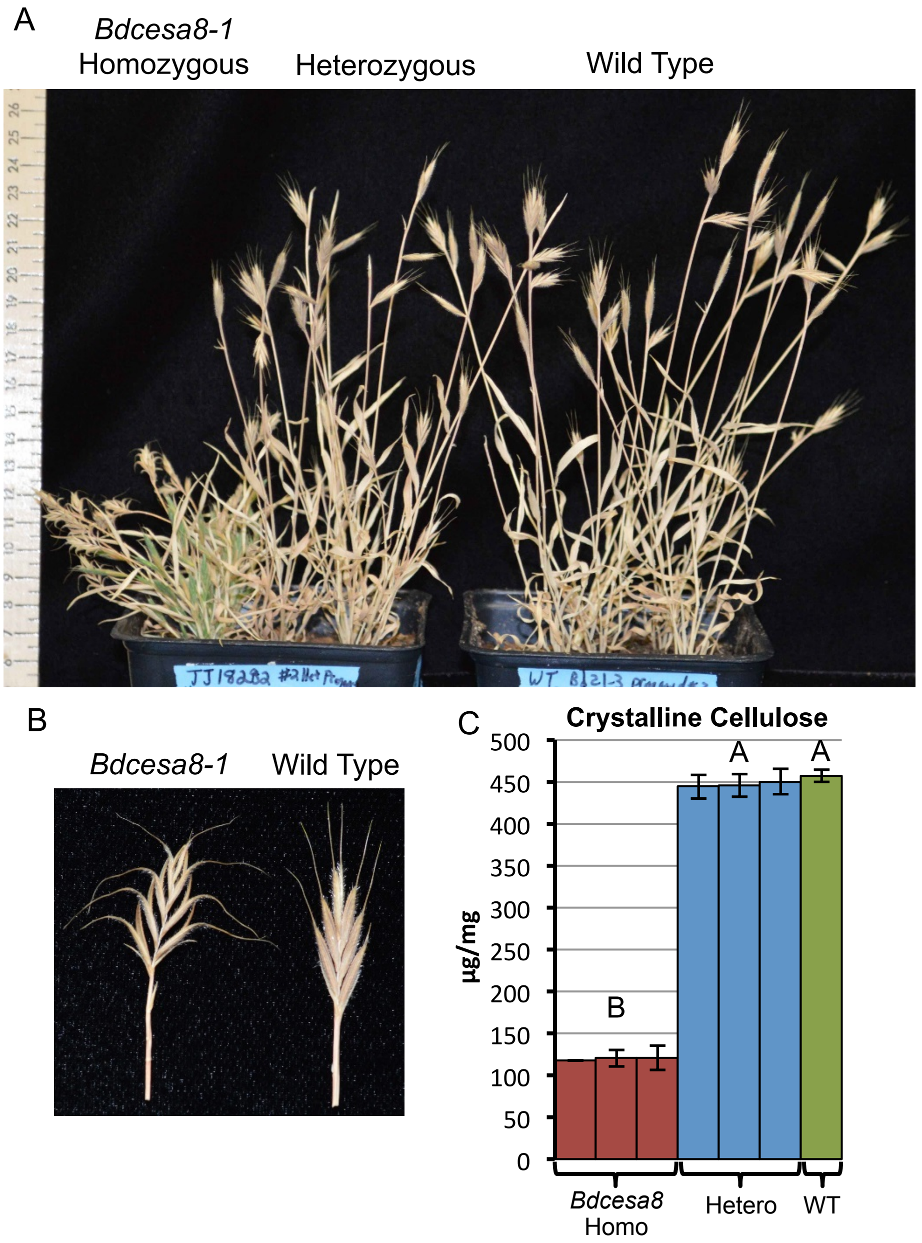

Supplemental Figure 12. *Bdcesa8-1* mutant culm growth and reduced cellulose phenotypes. **A)** Fully-grown homozygous *Bdcesa8-1*, heterozygous *BdCESA8*/*Bdcesa8-1*, and wild-type plants grown in 10 cm pots. Note the stunted growth of the homozygous *Bdcesa8-1* plants whereas the heterozygous plants look indistinguishable from wild type. **B)** Homozygous *Bdcesa8-1* (left) and wild-type (right) spikelets. Note the distorted awns and non-filled florets of the *Bdcesa8-1* spikelet. **C)** Crystalline cellulose content of senesced stems from the following plants: Homozygous *Bdcesa8-1*, heterozygous *BdCESA8*/*Bdcesa8-1* (hetero), and wild type (WT). Different letters represent statistically significant different means. Bars represent standard errors of three technical replicates.
